# Risk Stratification in Immunoglobulin A Nephropathy Using Network Biomarkers: Development and Validation Study

**DOI:** 10.2196/65563

**Published:** 2025-03-10

**Authors:** Jiaxing Tan, Rongxin Yang, Liyin Xiao, Lingqiu Dong, Zhengxia Zhong, Ling Zhou, Wei Qin

**Affiliations:** 1 Division of Nephrology, Department of Medicine West China Hospital of Sichuan University Chengdu China; 2 College of Computer Science Sichuan University Chengdu China; 3 Division of Nephrology, Department of Medicine Affiliated Hospital of Zunyi Medical University Zunyi China; 4 Division of Nephrology Zigong Third People's Hospital Zigong China

**Keywords:** IgA nephropathy, unsupervised learning, network biomarker, metabolomics, gut microbiota, biomarkers, risk stratification, IgA, immunoglobulin A, renal biopsy, renal, prospective cohort, Berger disease, synpharyngitic glomerulonephritis, kidney, immune system, glomerulonephritis, kidney inflammation, chronic kidney disease, renal disease, nephropathy, nephritis

## Abstract

**Background:**

Traditional risk models for immunoglobulin A nephropathy (IgAN), which primarily rely on renal indicators, lack comprehensive assessment and therapeutic guidance, necessitating more refined and integrative approaches.

**Objective:**

This study integrated network biomarkers with unsupervised learning clustering (k-means clustering based on network biomarkers [KMN]) to refine risk stratification in IgAN and explore its clinical value.

**Methods:**

Involving a multicenter prospective cohort, we analyzed 1460 patients and validated the approach externally with 200 additional patients. Deeper metabolic and microbiomic insights were gained from 2 distinct cohorts: 63 patients underwent ultraperformance liquid chromatography–mass spectrometry, while another 45 underwent fecal 16S RNA sequencing. Our approach used hierarchical clustering and k-means methods, using 3 sets of indicators: demographic and renal indicators, renal and extrarenal indicators, and network biomarkers derived from all indicators.

**Results:**

Among 6 clustering methods tested, the KMN scheme was the most effective, accurately reflecting patient severity and prognosis with a prognostic accuracy area under the curve (AUC) of 0.77, achieved solely through cluster grouping without additional indicators. The KMN stratification significantly outperformed the existing International IgA Nephropathy Prediction Tool (AUC of 0.72) and renal function-renal histology grading schemes (AUC of 0.69). Clinically, this stratification facilitated personalized treatment, recommending angiotensin-converting enzyme inhibitors or angiotensin receptor blockers for lower-risk groups and considering immunosuppressive therapy for higher-risk groups. Preliminary findings also indicated a correlation between IgAN progression and alterations in serum metabolites and gut microbiota, although further research is needed to establish causality.

**Conclusions:**

The effectiveness and applicability of the KMN scheme indicate its substantial potential for clinical application in IgAN management.

## Introduction

Immunoglobulin A nephropathy (IgAN) is the predominant primary glomerular disorder globally, presenting across diverse demographics. The prognosis of IgAN shows notable heterogeneity, with approximately 40% of patients progressing to end-stage renal disease, posing challenges for clinical judgment and management [1]. Hence, investigating risk factors for IgAN has been a recent research hotspot. Alongside traditional kidney-related markers such as proteinuria [2], hematuria [3], estimated glomerular filtration rate (eGFR) slope [4], uric acid [5,6] and Oxford classification [7], emerging extrarenal indicators like smoking status [8], bilirubin [9], platelet-albumin index [10], and triglyceride-glucose index [11] are increasingly explored for their prognostic value in IgAN. This suggests that clinicians should assess IgAN holistically, not just focusing on the renal biomarkers.

To assess IgAN’s prognosis, several stratification models have emerged, with the International Immunoglobulin A Nephropathy Prediction Tool (IIgAN-PT) by Barbour et al [12] being the most widely accepted. This model primarily focuses on traditional kidney-related indicators, such as the Oxford classification, treatment regimens, proteinuria, and blood pressure, and has been validated for prognostic accuracy by other research groups [13,14]. Recently, a Japanese cohort led by Koike et al [15] proposed the renal function–renal histology grading (RF-RG) stratification scheme for IgAN, primarily referencing the clinical grade (proteinuria and eGFR) and histological lesions to predict renal failure risk accurately, making it clinically promising due to its simplicity. Regrettably, these studies, while advancing IgAN risk stratification, are primarily retrospective, focusing mainly on traditional renal markers and often neglecting crucial extrarenal indicators. Consequently, these tools fail to differentiate prognosis among patients with similar renal damage who also have conditions like hepatitis B or diabetes—comorbidities that can impact outcomes. Moreover, while the classification schemes forecast prognosis risks, they provide minimal guidance for treatment, with IgAN guidelines largely relying on proteinuria, renal function, and limited histological data [16]. Therefore, current classification models for patients with IgAN are effective but need improvement.

Recent advancements in artificial intelligence, especially in unsupervised learning, have notably influenced various fields. This technique autonomously uncovers patterns and latent information within datasets, showing early successes in projects like gut microbiota classification and autism spectrum disorder stratification [17,18]. However, these applications often focus narrowly on isolated clinical metrics, missing broader interconnections. Simultaneously, the emergence of network biomarkers in precision medicine provides a deeper understanding of biological systems through the analysis of complex molecular interactions. This advanced approach improves our understanding of disease mechanisms, enhances prognostic accuracy, facilitates personalized treatments, and identifies critical stages in disease progression [19]. Yet, the potential of network biomarkers for clustering in biomedical research remains largely untapped, indicating a significant opportunity for further exploration and application in the field.

**Figure 1:**
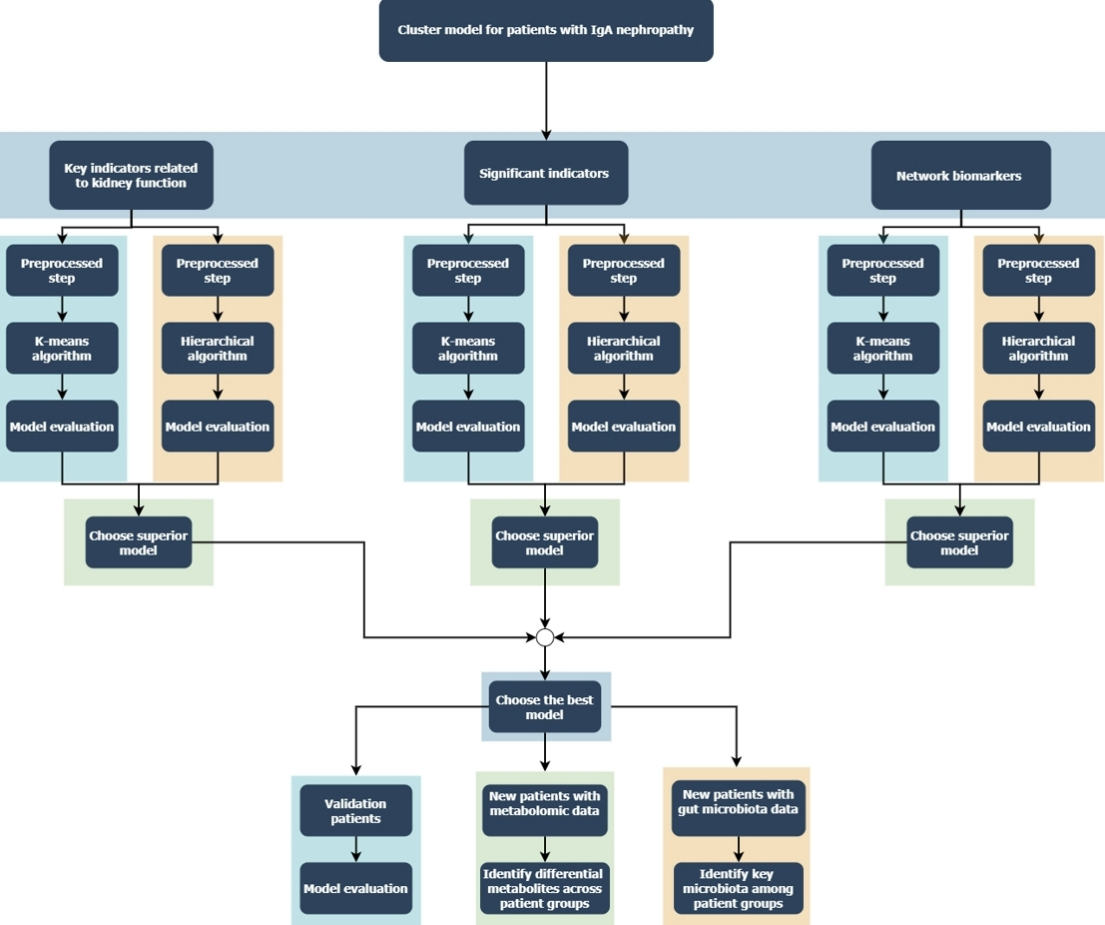
A comprehensive process for developing and applying clustering models. IgA: immunoglobulin A.

To address these issues, our study used a multicenter, prospective, observational cohort design to not only focus on traditional renal damage indicators but also incorporate extrarenal markers closely linked to the disease’s progression. By leveraging interdisciplinary techniques such as unsupervised learning and network biomarkers, we derived and externally validated optimal classification models for IgAN risk stratification. Additionally, we investigated whether these models could be used for long-term follow-up and to guide treatment decisions in IgAN. Furthermore, we examined significant differences in the metabolomics and gut microbiota under this stratification model, both of which have been documented in literature as closely related to the onset and progression of IgAN [20-26], aiming to provide preliminary clues into the mechanisms of IgAN progression (Figure 1).

## Methods

### Participant Recruitment

This multicenter, prospective cohort study included 1768 cases of IgAN diagnosed via renal biopsy between 2009 and 2021 at 4 nephrology centers: West China Hospital of Sichuan University, the Affiliated Hospital of Zunyi Medical University, Zigong Third People’s Hospital, and People’s Hospital of Mianzhu City. Follow-up data were last updated in October 2023 and are maintained by specialized technicians. The study included patients with a confirmed IgAN diagnosis and complete biopsy and clinical data, with IgAN identified as the primary disease. We excluded patients with insufficient pathological data, biopsies containing fewer than 8 glomeruli, incomplete follow-up data, or diagnosed with secondary IgAN. A total of 1460 patients from West China Hospital of Sichuan University meeting the inclusion criteria underwent unsupervised clustering, with the resulting algorithm subsequently validated on an independent cohort of 200 patients from other centers.

To assess the potential clinical meanings of this clustering, we analyzed separate groups of 63 and 45 patients who met our study criteria. These participants underwent ultraperformance liquid chromatography-mass spectrometry for serum metabolomic profiling and 16S RNA sequencing to identify differences in gut microbiota. Participants were free from metabolic diseases, such as hyperthyroidism and diabetes, and had no history of smoking, alcohol misuse, drug abuse, or infectious diseases. They had not received steroids or immunosuppressants in the past 6 months nor had they taken any lipid-lowering, uric acid–lowering, or metabolism-impacting drugs in the previous week. Additionally, they had not ingested probiotics, antibiotics, or gastrointestinal motility drugs in the last month and showed no significant digestive symptoms or diseases. The study adhered to the STROBE (Strengthening the Reporting of Observational Studies in Epidemiology) guidelines for observational studies, ensuring comprehensive and transparent reporting of methods and results.

### Ethical Considerations

Ethics approval for this study was obtained from the Ethics Committee of West China Hospital, Sichuan University (approval 2019-33), and all procedures strictly followed relevant ethical guidelines and regulations. Written informed consent was obtained from all participants, who were fully informed of their rights. Participant data were anonymized to ensure privacy and confidentiality, with securely stored, deidentified datasets accessible only to authorized researchers. No financial or material compensation was provided to participants. The study did not involve the inclusion of any identifiable individuals in images or Multimedia Appendix 1.

### Therapeutic Approaches and End Point

Adhering to KDIGO (Kidney Disease: Improving Global Outcomes) guidelines, our collaborative treatment approach between physicians and patients consists of 3 main protocols: supportive care with angiotensin-converting enzyme inhibitors (ACEIs) or angiotensin receptor blockers (ARBs), glucocorticoid administration starting at 0.5-1 mg/kg of prednisone with subsequent tapering, and a regimen of immunosuppressive agents, used with or without additional glucocorticoids [16]. Considering medication effects on the immune system, we merged glucocorticoid administration and immunosuppressive agents into immunosuppressive therapy (IST). The study aimed to evaluate composite outcomes, including end-stage renal disease, kidney transplantation, mortality, or significant declines in eGFR exceeding 50%.

### Data Processing and Unsupervised Clustering

We conducted our analysis using k-means and hierarchical clustering, following data preprocessing that involved imputing missing values with a multilinear interpolation algorithm, standardizing data to zero mean and unit variance, and reducing dimensionality with uniform manifold approximation and projection [27,28]. The k-means algorithm clusters data by first randomly assigning centroids and then iteratively relocating them to minimize intracluster variance, stabilizing when centroid movement ceases. Hierarchical clustering generates a dendrogram through agglomerative or divisive strategies: agglomerative starts with individual points as clusters, merging the most similar until one remains, while divisive begins with a single cluster that is continually split until each data point is isolated.

### Network Biomarkers

We conducted a perturbation analysis using a single sample-specific network approach to analyze individual samples, comparing their attributes with a reference group of 50 healthy individuals to identify distinctive features. Using graph feature engineering techniques, we extracted attributes at 3 network levels: node, subgraph, and global graph, to gain comprehensive insights. Node-level analysis included degree analysis to assess connectivity. At the subgraph level, we evaluated significant features like diameter and average clustering coefficient to identify disease-relevant patterns. At the global level, we analyzed node and edge counts and the number of connected components. Using the *Networkx* package (version 2.2) in Python (version 3.1.2; Python Software Foundation), we extracted 46 attributes for each sample’s network, which were used for clustering analysis [19,29,30].

### Interpretability Assessment of Unsupervised Clustering Algorithms

Unsupervised clustering algorithms, while powerful in detecting latent patterns, often lack interpretability. To address this issue, the subgroups identified by these algorithms were used as surrogate labels to develop a predictive framework through machine learning. This framework was refined using 10-fold cross-validation. An interpretability analysis was subsequently conducted on the proficient machine learning models using the Shapley Additive Explanations method, aiming to elucidate key indicators influencing classification outcomes [31].

### External Validation and Subsequent Omics Validation Labels

We applied data standardization and uniform manifold approximation and projection dimensionality reduction to the entire dataset rather than individual data points, using the patient cohort from the cluster set as the reference dataset. Each new patient was then processed sequentially using the standardization and dimensionality reduction models that were initially trained on this cluster set. After dimensionality reduction, the clustering model—previously trained on the cluster set—was used to assign a corresponding cluster label to each patient. This methodology ensures consistent treatment of new data within the established framework.

### Processing for Metabolomics Analysis

After fasting for at least 8 hours, about 5 mL of blood was drawn into anticoagulant-free vacutainers in the morning. These were left at room temperature to clot for 30 minutes before centrifuging at 3000 rpm for 10 minutes. The supernatant was then transferred to 1.5-mL Eppendorf tubes and stored at –80 °C for analysis.

Samples were thawed and prepared for ultraperformance liquid chromatography-mass spectrometry analysis, involving protein removal and reconstitution in a specific solvent. Hydrophilic interaction liquid chromatography was performed using a precise column and electrospray ionization for detection in both ionization modes. Mass spectrometry data were processed to identify peaks and extract ion features, with quality control measures to assess the variability of each metabolite, excluding those with a coefficient of variation over 20%. After sum normalization, data analysis included partial least-squares discrimination (PLS-DA) and calculation of variable importance in projection (VIP) values for each metabolite, considering VIP values >1 as statistically significant.

### Processing of Gut Microbiota Data

Approximately 4 g of fresh fecal samples were collected in sterile containers and stored at –80 °C for high-throughput sequencing. DNA was extracted from the samples, and its quantity and purity were assessed. The 16S ribosomal ribonucleic acid (rRNA) V3-V4 hypervariable regions were then amplified using specific primers and sequenced on the Illumina MiSeq platform. Raw sequencing reads were quality-filtered and clustered into operational taxonomic units at a 97% similarity threshold using UPARSE. The 16S rRNA sequences were classified using the ribosomal database project classifier algorithm against the Silva (SSU123) 16S rRNA database. Quality-controlled and standardized abundance data were used for subsequent analysis at the genus level.

### Statistical Analysis

Survival curves were generated using the Kaplan-Meier method, and differences between groups were assessed using the log-rank test. To estimate hazard ratios (HRs) and their 95% CIs, Cox proportional hazards models were used, with *P* values less than .05 indicating statistical significance. Additionally, extreme gradient boosting models were used to explore the associations between cluster labels and primary outcomes, with model performance evaluated by the area under the receiver operating characteristic curves. The standardized effect size differences among subtypes were quantified using Cohen *d*. Correlation network figures were constructed based on Spearman or Pearson correlation analyses to examine interactions between various metabolites and gut microbiota; edges were added to the network for any correlation with a *P* value below .05. Furthermore, Mantel tests were applied to analyze the associations between groups of clinical indicators and either metabolites or gut microbiota.

## Results

### Enhanced Performance of K-Means Clustering With Network Biomarkers in IgAN Stratification Over Other Unsupervised Learning Approaches

We curated a cohort of 1460 patients from West China Hospital of Sichuan University, strictly adhering to stringent inclusion criteria for our clustering set. The average follow-up period was 58.8 (SD 28.8) months. Our comprehensive dataset included demographics, clinical records, pathology, and treatment details such as age, blood pressure, serum creatinine, eGFR, 24-hour urine protein, urine red blood cell count, uric acid, renal immunofluorescence markers, Oxford MESTC classification (M, S, E, T, C), global and arteriolar sclerosis, lifestyle factors (smoking and drinking), liver function, comorbidities (diabetes and hepatitis B), and lipid metabolism markers.

**Figure 2:**
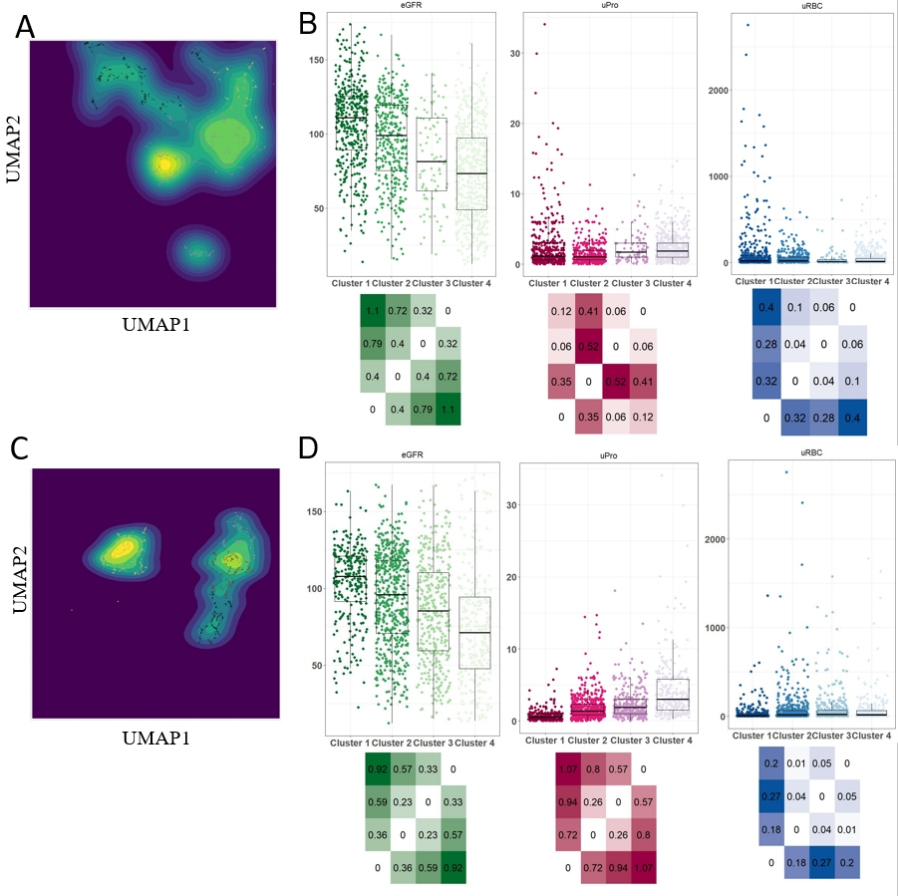
Clustering based on unsupervised learning with renal and extrarenal indicators and network biomarkers. (A) Visual representation of UMAP-reduced datasets integrated with k-means clustering, focusing on renal and extrarenal indicators. Each individual is stratified by subtype and depicted through density plots, highlighting the distribution within each group. (B) Analysis of the subtypes derived from the methodologies described in (A), presented using scatter boxplots and heatmaps. These illustrate the pairwise standardized effect size differences (Cohen d) between subtypes, facilitating a clear comparison of indicator levels across groups. (C) Visual representation of UMAP-reduced datasets combined with k-means clustering, focusing on network biomarkers. Individuals are stratified by subtype and depicted through density plots to showcase the clustering outcome. (D) Analysis of the subtypes derived from the methodologies described in (C), presented using scatter boxplots and heatmaps. These visualizations detail the pairwise standardized effect size differences (Cohen d) between subtypes, providing insights into the variation of network biomarkers across different groups. eGFR: estimated glomerular filtration rate; UMAP: uniform manifold approximation and projection; uPro: urine protein; uRBC: urine red blood cell count.

We used hierarchical clustering and k-means methods, which are widely used in the medical field for unsupervised clustering [17,32,33]. These methods used 3 sets of indicators: the first set included demographic and renal indicators, the second set combined renal and extrarenal indicators, and the third set comprised network biomarkers derived from all indicators. The objective was to explore whether classifications based on a broader range of systemic markers could outperform those focusing solely on renal indicators, aiming to address potential shortcomings in existing stratification schemes and guidelines. Network biomarker construction was based on references from our prior research [29]. All 6 clustering methods successfully categorized all patients with IgAN into 4 groups, as demonstrated in Figure 2 and Figures S1-S4 in Multimedia Appendix 1. As cluster numbers rose from 1 to 4, symptom severity increased, reflecting closely with clinical evaluations, as evidenced by declining mean eGFR values and rising mean proteinuria values. Although clinical intuition enabled physicians to categorize patients using all indicators, this experience-based and direct judgment alone was not consistently accurate in dividing patients into 4 groups, especially regarding cluster 2 and cluster 3.

**Figure 3:**
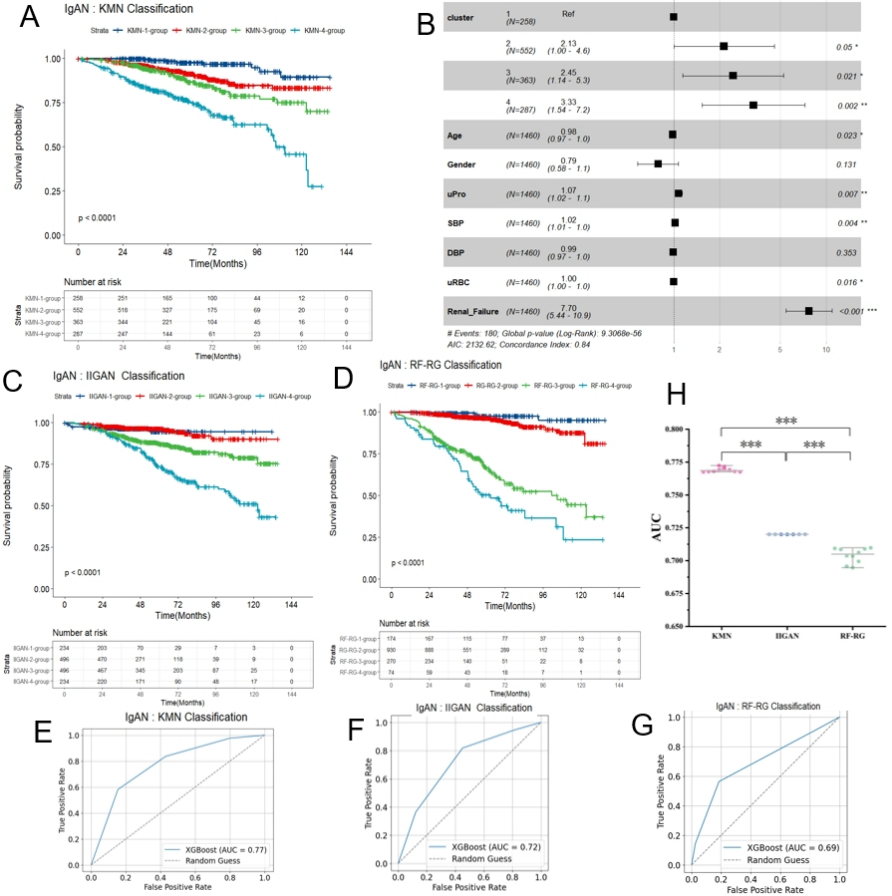
Survival analysis comparisons across different classification methods for IgAN. (A) Kaplan-Meier curves illustrating mortality rates in IgAN, stratified by KMN grouping. (B) Forest plot illustrating the results of a multifactor Cox regression analysis, stratified by KMN grouping. (C). Kaplan-Meier curves showing IgAN mortality rates, stratified by IIGAN grouping. (D) Kaplan-Meier curves detailing IgAN mortality rates, stratified by RF-RG grouping. (E) ROC curve for the KMN grouping, with an AUC value of 0.77. (F) ROC curve for the IIGAN grouping, featuring an AUC value of 0.72. (G) ROC curve for the RF-RG grouping, with an AUC value of 0.69. (H) Comparison of AUCs for classification schemes in IgAN risk stratification. *P<.05, **P<.01, ***P<.001. AIC: Akaike information criterion; AUC: area under the curve; DBP: diastolic blood pressure; IgAN: immunoglobulin A nephropathy; IIGAN: International Risk-Prediction Tool in Immunoglobulin A Nephropathy; KMN: k-means clustering based on network biomarkers; RF-RG: renal function–renal histology grading; ROC: receiver operating characteristic; SBP: systolic blood pressure; uPro: urine protein; uRBC: urine red blood cell count; XGBoost: extreme gradient boosting.

Unsupervised clustering not only accurately differentiated patients but also proved highly effective in evaluating IgAN prognosis. Among the 6 methods used, 3 stood out for effectively reflecting IgAN prognosis: hierarchical clustering based on renal indicators, k-means clustering based on all indicators, and k-means clustering based on network biomarkers (KMN). Kaplan-Meier curves for the 4 clusters showed significant differences (*P*<.0001; Figure 3A and Figure S5A and B in Multimedia Appendix 1), indicating their prognostic relevance, as supported by a multivariable Cox regression model adjusted for age, gender, urine protein, urine red blood cells, renal function, and blood pressure (*P*<.0001; Figure 3B and Figure S3C and D in Multimedia Appendix 1). Figure S6 in Multimedia Appendix 1 presents further prognostic parameters, reinforcing previous findings: notably, cluster 1 has the best prognosis, whereas cluster 4 has the worst, consistent with clinical categorizations.

We evaluated the effectiveness of various clustering methods in reflecting IgAN prognosis solely by using cluster labels as input variables and assessing the area under the curve (AUC). This approach holds significant value in assessing the predictive performance of the model. Our study found that KMN exhibited the highest AUC at 0.77 (Figure 3E), outperforming hierarchical clustering based on renal indicators (0.71; Figure S5E in Multimedia Appendix 1) and k-means clustering based on all indicators (0.75; Figure S5F in Multimedia Appendix 1), indicating superior predictive power for KMN and surpassing methods that rely solely on renal indicators by 0.6. Therefore, the k-means clustering approach, based on network biomarkers, was recommended for stratifying IgAN risk.

**Figure 4:**
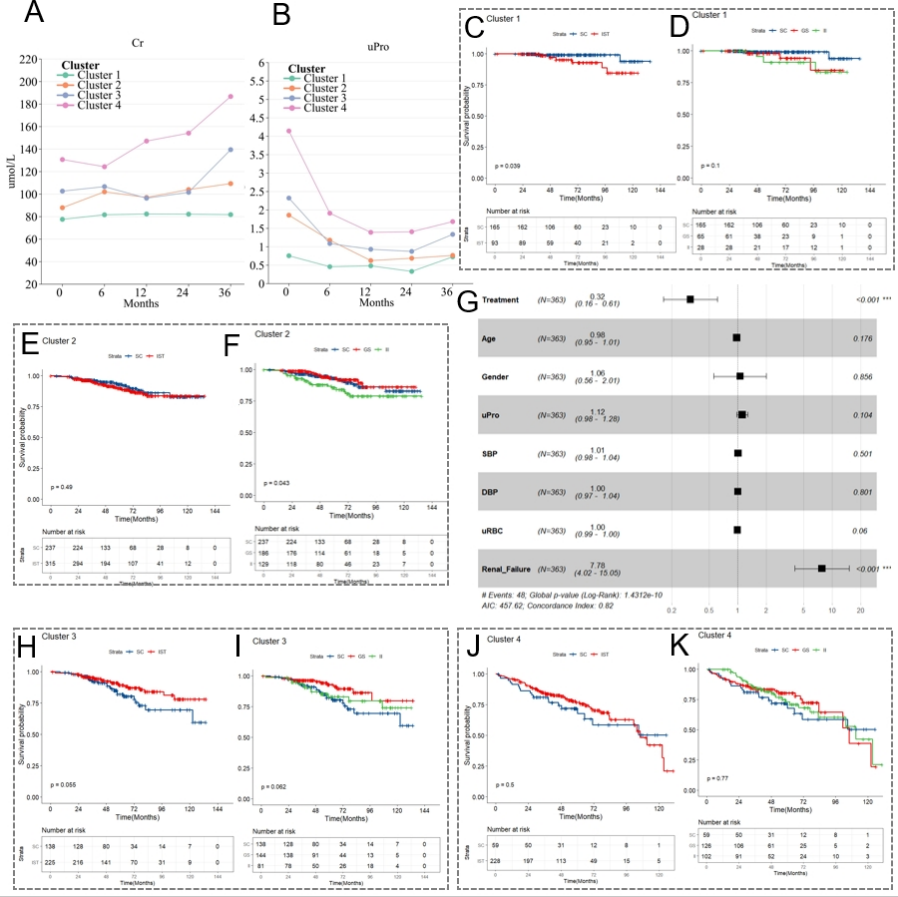
Tracking 3-year renal indicators in immunoglobulin A nephropathy and guiding treatment based on KMN grouping. (A) Line graphs depicting the average serum Cr levels of patients in each KMN group over time, illustrating changes in kidney function. (B) Line graphs showing the average uPro levels for each KMN group over time, highlighting trends in proteinuria across different clusters. (C) Kaplan-Meier curves for cluster 1 patients, stratified by treatment type: SC and IST, which includes steroids and other IIs. (D) Kaplan-Meier curves for cluster 1, detailing survival outcomes under different treatments: SC, GS, and IIs. (E and F), (H and I), and (J and K) Kaplan-Meier curves representing survival outcomes for clusters 2, 3, and 4, respectively, under various treatment modalities. (G) A forest plot visualizing the results of a multifactor Cox regression, specifically stratified by the third KMN grouping. ***P<.001. Cr: creatinine; DBP: diastolic blood pressure; GS: glucocorticoid therapy; II: immunosuppressive agents; IST: immunosuppressive therapy; k-means clustering based on network biomarkers; SBP: systolic blood pressure; SC: supportive care; uPro: urine protein; uRBC: urine red blood cell count.

To further investigate the impact of the KMN stratification on the prognosis of IgAN, we assessed the 3-year trajectories of urinary protein and serum creatinine across different patient groups. Figure 4A and B and Figures S7 and S8 in Multimedia Appendix 1 demonstrate that most patients in cluster 1 exhibited relatively stable average urinary protein levels and normal fluctuations in creatinine levels. Patients in cluster 2 had higher baseline levels of urinary protein and creatinine than those in cluster 1 but exhibited a declining trend in urinary protein over time. Meanwhile, their creatinine levels increased slightly, with most remaining within chronic kidney disease stages 1, 2, or 3a by the third year. In cluster 3, patients experienced a reduction in urinary protein at the 6-month mark compared to baseline but maintained higher levels (around or above 1 g in 24 hours) throughout the 3 years, with both creatinine and urinary protein showing significant increases by the third year, indicating a potential rapid deterioration of their condition. Patients in cluster 4, having the most severe prognosis, showed a consistent yearly increase in creatinine, quickly progressing toward renal failure, and maintained high levels of urinary protein. These findings suggested that patients in different KMN stratifications had distinct trajectories, indicating that KMN stratification could serve as an effective prognostic marker for IgAN.

### Superiority of KMN Stratification Over IIgAN-PT and RF-RG Schemes

To determine if our KMN scheme outperforms the globally used renal marker–based IIgAN-PT and RF-RG schemes [12,15], we first assessed the applicability of IIgAN-PT and RF-RG to our cohort. As shown in Figure 3C and D, the stratification of our cohort into 4 groups using RF-RG and IIgAN-PT classification methods clearly demonstrates significant disparities in the Kaplan-Meier curves (*P*<.0001), confirming the prognostic utility of these schemes for our cohort. Multivariable Cox regression analysis (Figure S9 in Multimedia Appendix 1) revealed that in IIgAN-PT, compared to cluster 1, the HRs for clusters 2, 3, and 4 were 0.79 (95% CI 0.35-1.79), 1.69 (95% CI 0.80-3.59), and 3.03 (95% CI 1.41-6.53), respectively. Similarly, for RF-RG, the HRs for clusters 2, 3, and 4 were 2.28 (95% CI 0.81-6.42), 6.60 (95% CI 2.08-20.96), and 8.54 (95% CI 2.55-28.63), respectively. These results suggest that all 6 of our clustering approaches outperformed the RF-RG and IIgAN-PT schemes. Considering potential collinearity between the primary indicators used in these models and the covariates adjusted in our Cox regression, we primarily assessed the models using AUC values. The AUC values for IIgAN-PT and RF-RG were 0.72 (Figure 3F) and 0.69 (Figure 3G), respectively, indicating that IIgAN-PT performs better than RF-RG but is inferior to our KMN model, which achieved an AUC of 0.77 (Figure 3E). We conducted 10 rounds of random sampling to generate AUC values for each classification scheme. A Mann-Whitney *U* test on the resulting AUC values revealed that the *P* values for differences between any 2 classification schemes were less than .001. These results confirm statistically significant differences between the methods, with our clustering approach outperforming both RF-RG and IIgAN-PT (Figure 3H).

### External Validation of KMN Stratification

To assess whether our risk stratification could be applied to external cohorts, we conducted further validation by including 200 patients with IgAN who met the inclusion and exclusion criteria from the Affiliated Hospital of Zunyi Medical University, Zigong Third People’s Hospital, and People’s Hospital of Mianzhu City. Our findings indicated that the KMN-based stratification effectively categorized patients in these additional cohorts, with clinical symptoms and predictive performance consistent with previous results (Figure S10 in Multimedia Appendix 1).

### Guidance of Treatment Decisions by KMN Stratification Scheme

The current treatment for IgAN predominantly relies on ACEIs or ARBs, with the option of IST for more severe cases. The KIDGO guidelines focus mainly on creatinine and urinary protein levels, offering less guidance on pathological indices or systemic status [16], and do not provide specific recommendations for therapies under the IIgAN-PT or RF-RG schemes [12,15]. Our study aimed to determine if our KMN stratification could effectively guide immunosuppressive treatment for IgAN, a topic currently under debate [34-36]. While recent studies have introduced new therapeutic options such as atacicept [37], telitacicept [38], sparsentan [39], irbesartan [40], and dapagliflozin [41], these were not used in our cohort, which primarily consists of patients followed for over 3-5 years before these drugs were fully available. We explored the utility of KMN stratification in guiding IST through Cox multivariable regression and survival curve analyses as shown in Figure 4 and Figure S11 in Multimedia Appendix 1.

The Kaplan-Meier curve shows significant differences in survival rates within cluster 1 between supportive care, primarily using ACEIs or ARBs, and various ISTs including glucocorticoids and other immunosuppressive agents, underscoring that these therapies might not confer any additional benefit for these patients.

In cluster 2, there was no significant difference between IST and supportive care (*P*=.49), with supportive care and glucocorticoids performing similarly and better than immunosuppressive agents (*P*=.04). Given the significant side effects of glucocorticoids and the negative results from multivariable Cox regression for immunosuppressants, supportive care remains the preferred treatment for this group.

For cluster 3, glucocorticoids were found to slightly outperform other immunosuppressants, which themselves were marginally better than supportive care alone (*P*=.06). Combining glucocorticoids and immunosuppressants demonstrated a clear advantage over supportive care (*P*=.05). Multivariable Cox regression (Figure 4G) revealed a significant renal protective effect of this combined IST, with an HR of 0.32 (95% CI 0.16-0.61). Given the data showing poor prognosis and rapid renal deterioration typically within 3 years for cluster 3 patients, we strongly recommend immunosuppressive treatment, favoring glucocorticoids or, if intolerable, alternative immunosuppressants such as mycophenolate mofetil.

For cluster 4, which had the poorest prognosis, survival curves and Cox regression analyses showed no significant differences between immunosuppressive treatment and supportive care. However, upon closer examination, immunosuppressive treatment outperformed supportive care for up to 96 months, with intersections thereafter likely due to the small number of patients followed for this duration, which could explain the absence of statistical significance. Given the severity of their condition, immunosuppressive treatment should be considered for these patients if they could manage the side effects, with decisions tailored to individual circumstances.

### Interpretable Analysis of KMN Stratification

Unsupervised clustering based on network biomarkers exhibited a “black box” nature; while effective in assessing clinical severity, prognosis, and treatment, it lacked intuitive interpretation. Therefore, we conducted an innovative interpretive analysis of the unsupervised clustering approach, which might offer additional insights for clinical practice. Figure S12 in Multimedia Appendix 1 highlights the key factors influencing the KMN classification, revealing significant network parameters such as diameter, nodes, and edges. Notably, the networks centered on urinary protein, red blood cells, age, and eGFR were particularly crucial for stratifying IgAN. We further analyzed the networks centered on these parameters for patients in clusters 1-4, discovering significant differences among these groups (Figures S13-S16 in Multimedia Appendix 1). This suggested that not only did urinary protein, urinary red blood cells, age, and eGFR individually impact the prognosis of IgAN, but the relationships between these markers and other indicators also significantly correlated with outcomes. This aspect was often overlooked by traditional statistical methods, which had primarily focused on isolated indicators, especially renal-related ones, neglecting the statistical significance of these networks.

### Metabolomic Diversity Analysis Based on KMN Stratification

**Figure 5:**
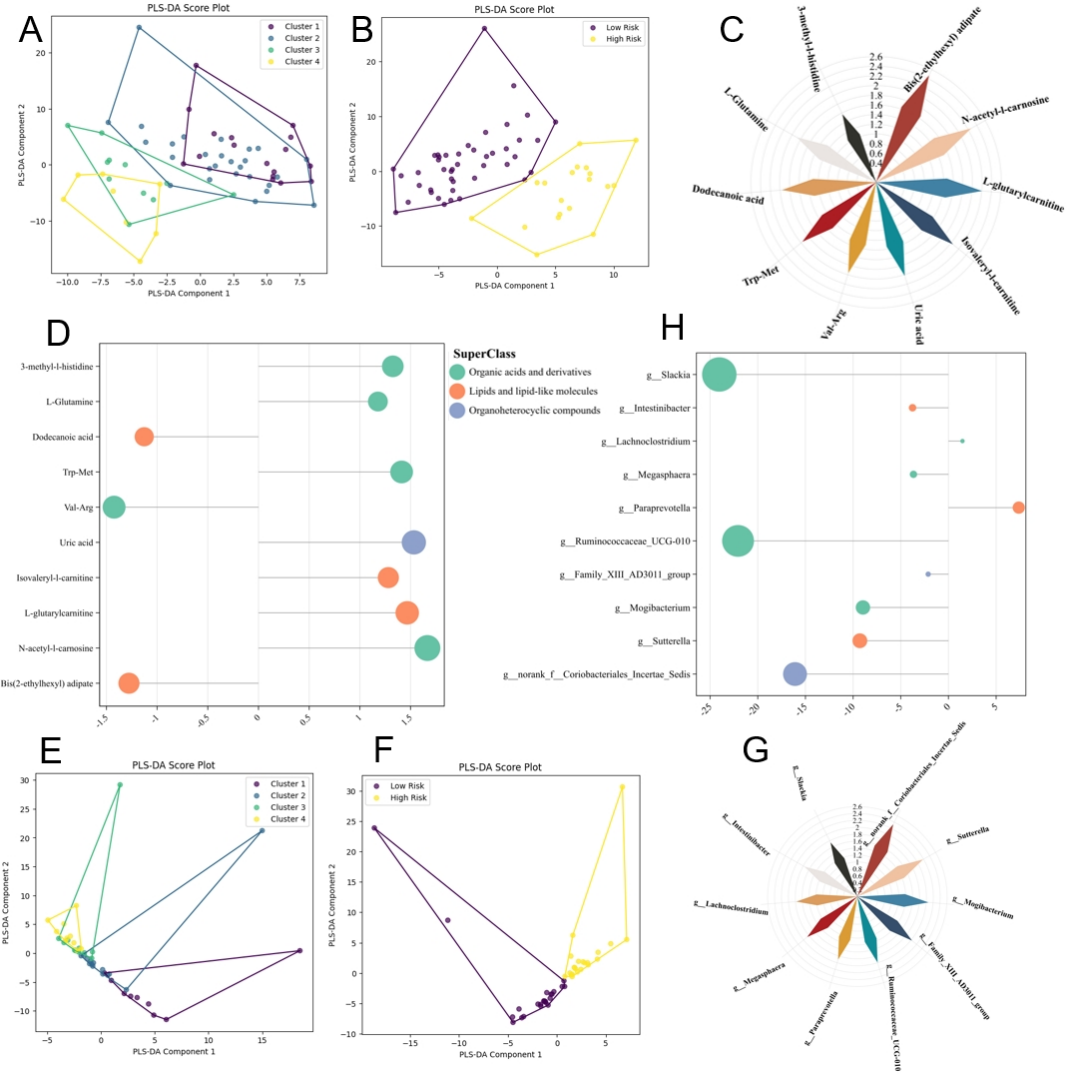
Differentiation of serum metabolites and gut microbiota in IgAN progression revealed by KMN grouping. (A) PLS-DA score plots vividly illustrating the separation trends of serum metabolites among the 4 KMN clusters (1, 2, 3, and 4). (B) PLS-DA analysis highlighting significant structural differences in serum metabolites between the high-risk IgAN groups (clusters 3 and 4) and low-risk IgAN groups (clusters 1 and 2). (C) Windrose visualization of variable importance in projection values for metabolites across the 4 grouped PLS-DA models, demonstrating the impact of key metabolites in differentiating the clusters. (D) Bar graph showing the fold changes of differential metabolites in high-risk groups compared to low-risk groups, indicating their increase or decrease in abundance. (E) PLS-DA score plots vividly showcasing the separation trends of gut microbiota among the 4 KMN clusters. (F) PLS-DA analysis indicating significant structural differences in gut microbiota between the high-risk IgAN groups and low-risk IgAN groups. (G) Windrose visualization of variable importance in projection values for gut microbiota across the 4 grouped PLS-DA models, highlighting the impact of key microbiota at the genus level in differentiating the clusters. (H) Bar graph illustrating the fold changes of differential gut microbiota on the genus level in high-risk groups compared to low-risk groups, showing their increase or decrease in abundance. IgAN: immunoglobulin A nephropathy; KMN: k-means clustering based on network biomarkers; PLS-DA: partial least-squares discrimination.

To identify potential serum metabolomic markers for the progression of IgAN, we used the KMN method to stratify 63 new patients with blood metabolomics data into 4 clusters: cluster 1 with 17 individuals, cluster 2 with 27, cluster 3 with 11, and cluster 4 with 8. After quality control and normalization of the metabolomics data, we conducted a PLS-DA model analysis and observed a distinct separation trend among the 4 clusters (Figure 5A) [42]. Clusters 1 and 2 were then reclassified into a new cohort labeled the low-risk group, and clusters 3 and 4 into a high-risk group. Further PLS-DA analysis of the metabolomic data revealed significant disparities between the high-risk and low-risk classifications (Figure 5B). Additionally, we constructed metabolite interaction networks for the clusters, uncovering distinct intermetabolite relationships across them (Figure S17 in Multimedia Appendix 1). These findings indicated that the differences in serum metabolites between various risk levels of IgAN were significant and that changes in their interaction networks might also contribute to the disease’s progression.

Through VIP analysis of the PLS-DA models for the 4 clusters, we identified the top 10 metabolites that most significantly differentiate the clusters (Figure 5C and D). Notably, levels of bis(2-ethylhexyl) adipate (3-dehydroepiandrosterone sulfate [DEHA]), Val-Arg, and dodecanoic acid were reduced in the high-risk group. DEHA, a plasticizer not typically found in the human body and primarily ingested externally, has no well-documented links to renal diseases, and its metabolic pathways in the body remain unclear. Some studies suggest DEHA could have carcinogenic effects, cause DNA damage, and disrupt steroidogenesis pathways [43-46]. We hypothesize that in high-risk IgAN, abnormal kidney function could decrease the renal excretion of microplastics, leading to their uptake by cells, potentially causing cellular damage, though further experimental evidence is needed to confirm this. Additionally, Val-Arg, a dipeptide involved in protein metabolism, and dodecanoic acid, a medium-chain fatty acid, may indicate disruptions in protein and fatty acid metabolism in high-risk patients.

Conversely, levels of N-acetyl-L-carnosine (a dipeptide with antioxidant properties) [47], L-glutarylcarnitine and isovaleryl-L-carnitine (both involved in fatty acid metabolism) [48,49], uric acid, Trp-Met (a tryptophan and methionine complex indicating protein metabolism), L-glutamine (an amino acid vital for immune function) [50,51], and 3-methyl-L-histidine (a marker of muscle metabolism) [52] were elevated in the high-risk group, pointing to enhanced oxidative stress and altered protein and energy metabolism in these patients.

An interesting finding from our study was the strong correlation among metabolites, which suggested that their collective interactions might influence the severity of IgAN and potentially lead to poor outcomes (Figure S18A in Multimedia Appendix 1). Additionally, by focusing on these metabolites, we explored the metabolite networks across different clusters and observed distinct network patterns between them (Figures S19-S25 in Multimedia Appendix 1). These variations in metabolite levels and their interactions could provide insights into the mechanisms underlying IgAN progression, particularly in high-risk individuals.

Further investigation into the relationship between these metabolites and clinical indicators revealed that, except for Val-Arg, which demonstrated a statistical correlation with kidney function, the levels of other metabolites did not correlate with traditional renal function markers such as urinary protein, red blood cells, and pathological lesions. This finding suggests that classifications based solely on renal function markers might miss crucial metabolites that play a significant role in the disease’s progression. Therefore, our KMN stratification offers a novel approach to understanding the complex mechanisms driving IgAN progression, highlighting the importance of broader biomarker profiles in disease analysis.

### Gut Microbiota Diversity Analysis Based on KMN Stratification

Similarly, we recruited an additional 45 patients, stratified into 4 clusters using the KMN method: 9 individuals in cluster 1, 14 in cluster 2, 12 in cluster 3, and 10 in cluster 4. We conducted gut microbiome sequencing on these individuals. Subsequent PLS-DA model analysis of the reclassified cohorts revealed significant disparities between the high- and low-risk groups (Figure 5E and F), indicating substantial differences in the gut microbiomes among patients with varying risk levels. Additionally, the analysis of microbiome interaction networks (Figure S29 in Multimedia Appendix 1) clearly showed distinct intermicrobial relationships across the clusters, suggesting that changes in these interactions may be related to disease progression.

Through VIP analysis of the PLS-DA models for the 4 clusters, significant variances in gut microbiota were identified, delineating distinct profiles between high- and low-risk groups (Figure 5G and H). Elevated levels of *Paraprevotella* and *Lachnoclostridium*—the latter closely linked to kidney function (Figure S18B in Multimedia Appendix 1)—in the high-risk group suggest their role in exacerbating IgAN through gut dysbiosis, given their association with inflammatory processes [53,54]. Conversely, decreased levels of norank_f_Coriobacteriales_Incertae_Sedis, *Sutterella*, *Mogibacterium*, Family_XI AD3011_group, Ruminococcaceae_UCG-010, *Megasphaera*, *Intestinibacter*, and *Slackia* were noted. These genera might be involved in fermenting dietary fibers and producing short-chain fatty acids, essential for colon health and immune modulation [55,56]. Their reduced presence might suggest a loss of protective microbial functions in patients at higher risk of disease progression. Additionally, the interaction patterns between these microbiota batches underscore the potential of gut bacteria as biomarkers for stratifying IgAN risk and highlight the complex relationship between gut health and kidney disease (Figures S26-S34 in Multimedia Appendix 1). However, these causal relationships need to be substantiated by further studies.

## Discussion

### Principal Findings

Our study used network biomarkers combined with unsupervised clustering to develop a novel stratification scheme for IgAN, offering distinct advantages over existing approaches. By integrating a broad array of variables, including but not limited to renal indicators—such as liver function, lipid metabolism, comorbidities, and lifestyle factors—our method offers a more holistic assessment of patients. Additionally, we examined the interrelationships among these indicators, applying clustering techniques to their interaction vectors, which may more accurately reflect biological interactions. Our stratification scheme has undergone external validation, indicating potential applicability to diverse cohorts. Clinically, this stratification could assist in personalizing treatment, suggesting supportive ACEI or ARB treatment for patients in lower-risk groups (clusters 1 and 2) and potentially considering IST for those in higher-risk groups (clusters 3 and 4).

To our knowledge, our study was the first to integrate k-means clustering with network biomarkers. K-means clustering partitions patients into a predetermined number of clusters based on their similarity to cluster centroids, which is computationally efficient and well-suited for large datasets [32,57]. This makes it particularly effective for managing high-dimensional data in the classification of patients with IgAN. Network biomarkers, offering several advantages over traditional biomarkers, capture the intricate relationships between different biological factors and provide a nuanced understanding of disease heterogeneity [19,30]. They also offer a system-level view that highlights the interconnectedness of various physiological processes involved in IgAN pathogenesis.

By incorporating network biomarkers into k-means classification models, our method outperformed traditional molecular biomarker–based clustering in distinguishing varying degrees of clinical severity in IgAN. This approach not only more accurately categorized patients but also surpassed existing prognostic models, providing deeper insights into disease progression by our interpretable analysis. The innovative integration of network biomarkers enhances our ability to capture complex biological interactions and systemic effects that traditional markers might overlook. This improves the accuracy of IgAN prognosis and suggests the method’s applicability to other diseases with similar complex traits. The scalability and adaptability of this approach could significantly improve disease classification and management across various clinical cohorts, leading to more personalized and effective treatment strategies.

Previous metabolomic and gut microbiome studies have indicated structural changes in the metabolites and microbiota of patients with IgAN compared to healthy individuals [20,24,26]. However, clinical research exploring their impact on disease progression remains preliminary and simplistic. Most investigations into these substances have been conducted through animal or cell experiments to elucidate the changes in the gut-kidney axis and their influence on IgAN [22,24]. Building on this foundation, our study conducted further research, using the KMN stratification to more accurately predict clinical outcomes for these patients, moving beyond simple classifications based on proteinuria and creatinine. We observed significant differences in serum metabolite structures and gut microbiome compositions between high-risk and low-risk patients with IgAN. Importantly, the interaction networks between metabolites and microbiota also varied distinctly, suggesting that changes in these elements and their interactions are closely linked to the progression of IgAN. Preliminary clinical evidence from our study shows that a reduction in beneficial bacteria and an increase in potentially pathogenic microbes, along with environmental pollution, metabolic abnormalities in proteins and lipids, oxidative stress, and immune dysregulation, collectively contribute to the progression of IgAN [58,59]. These findings are consistent with other basic and clinical research, indirectly validating the clinical relevance of our KMN stratification.

Our study has several limitations that warrant discussion. First, our cohort consisted exclusively of Chinese individuals, lacking diversity in racial data. Given the heterogeneity of IgAN across different races, our existing classification scheme may not be directly applicable to other populations. However, other ethnic groups could potentially use our approach of unsupervised clustering based on network biomarkers to develop tailored classification schemes suited to their specific racial characteristics. Second, our analyses of the gut microbiome and metabolomics were essentially cross-sectional, which, despite using the KMN stratification scheme providing prognostic insights, does not establish causality. Additionally, the sample size was relatively small, meaning that our findings represented only a preliminary characterization of the gut microbiome and serum metabolites to validate the clinical value of our KMN stratification. Further in-depth studies are needed to explore the underlying mechanisms of these findings.

### Conclusions

This study developed a novel risk stratification model for IgAN using network biomarkers and unsupervised clustering. The KMN scheme outperformed existing models in predicting disease progression and guiding personalized treatment. Our findings also highlight the potential role of systemic biomarkers, including serum metabolites and gut microbiota, in IgAN management. Further research is needed to validate these insights and enhance clinical applicability.

## Appendix

Multimedia Appendix 1 Supplementary figures for evaluating the value of KMN stratification in distinguishing IgAN severity, prognosis, treatment guidance, and multi-omics analysis.

## Abbreviations

ACEI: angiotensin-converting enzyme inhibitor
ARB: angiotensin receptor blocker
AUC: area under the curve
DEHA: 3-dehydroepiandrosterone sulfate
eGFR: estimated glomerular filtration rate
HR: hazard ratio
IgAN: immunoglobulin A nephropathy
IIgAN-PT: International Immunoglobulin A Nephropathy Prediction Tool
IST: immunosuppressive therapy
KDIGO: Kidney Disease: Improving Global Outcomes
KMN: k-means clustering based on network biomarkers
PLS-DA: partial least-squares discrimination
RF-RG: renal function–renal histology grading
rRNA: ribosomal ribonucleic acid
STROBE: Strengthening the Reporting of Observational Studies in Epidemiology
VIP: variable importance in projection

## Data Availability

All primary data supporting our findings are provided in the main text and in Multimedia Appendix 1. Due to privacy and ethical considerations, secondary datasets generated or analyzed during this study are not publicly accessible. However, these datasets can be obtained from the corresponding author upon reasonable request, subject to strict adherence to privacy regulations and ethical guidelines approved by the ethics committee of West China Hospital, Sichuan University. The software code and methodologies used to derive patient labels are available in our GitHub repository, titled Unsupervised Learning for Risk Stratification in IgA Nephropathy Using Network Biomarkers [60]. This repository provides full transparency of our analytical techniques, facilitating further research and validation.
