# Supplementary material for: Risk Stratification in Immunoglobulin A Nephropathy Using Network Biomarkers: Development and Validation Study
Source: J Med Internet Res. 2025 Mar 10;27:e65563. doi: 10.2196/65563 (PMC11933752; doi:10.2196/65563)

**Figure S1**. A. Visual representations of UMAP-reduced datasets integrated into **hierarchical clustering analyses using renal indicators**. Individuals are stratified by subtype and depicted through density plots. B. Clarification of the derived subtypes, obtained through the aforementioned methodology, using scatter boxplots and heatmaps to depict the pairwise standardized effect size difference (Cohen’s d) between these subtypes.


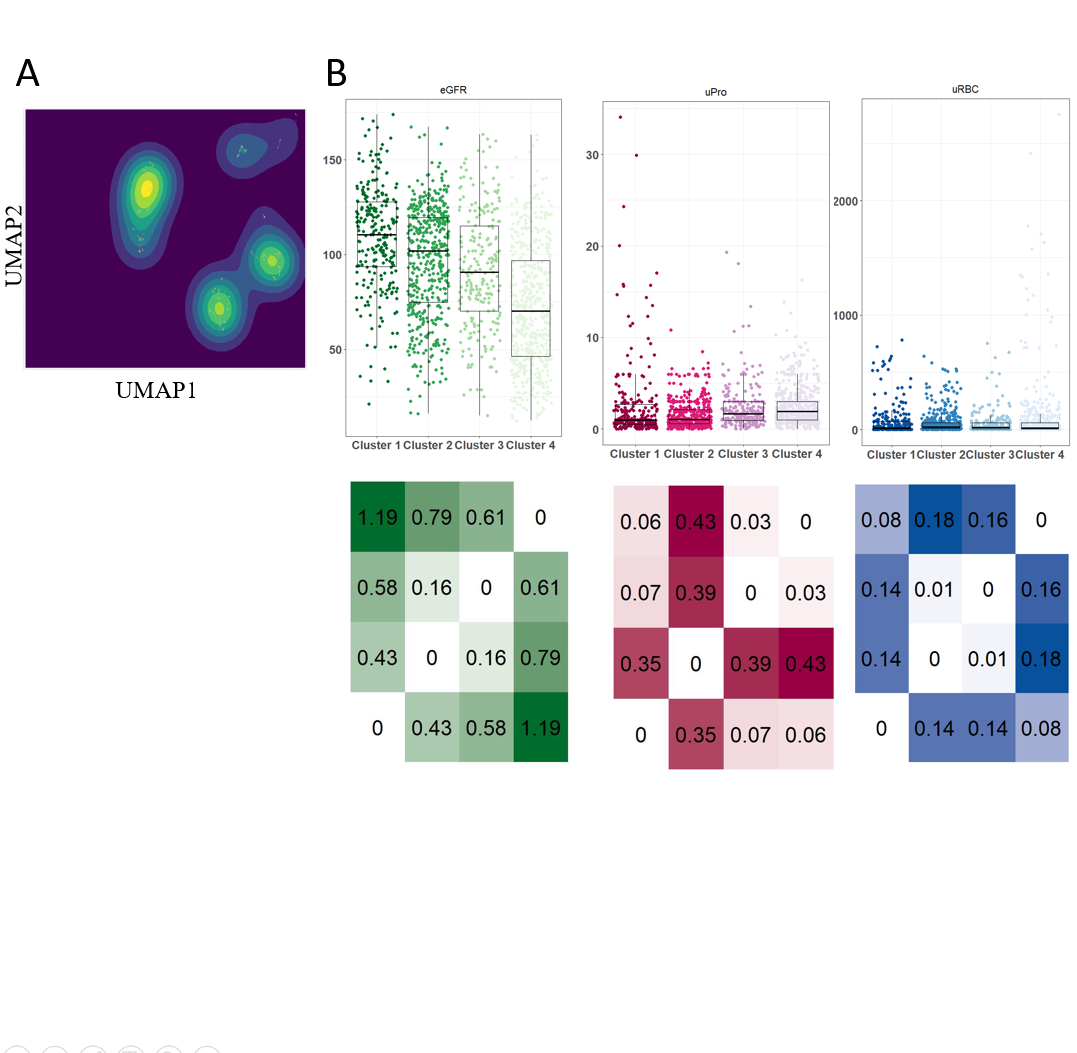


**Figure S2**. A. Visual representations of UMAP-reduced datasets integrated into **k-means analyses using renal indicators**. Individuals are stratified by subtype and depicted through density plots. B. Clarification of the derived subtypes, obtained through the aforementioned methodology, using scatter boxplots and heatmaps to depict the pairwise standardized effect size difference (Cohen’s d) between these subtypes.


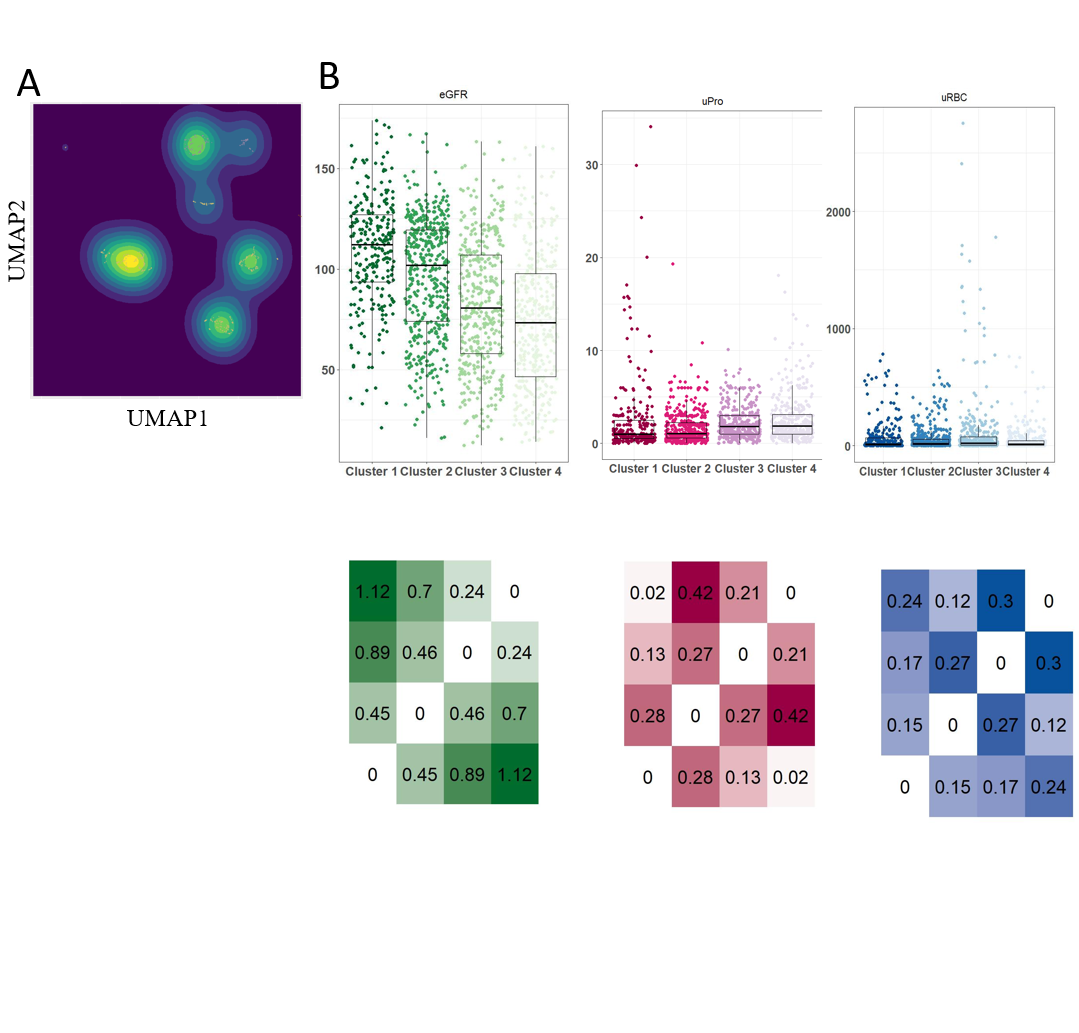


**Figure S3.** A. Visual representations of UMAP-reduced datasets integrated into **hierarchical clustering analyses using both renal and extra-renal indicators**. Individuals are stratified by subtype and depicted through density plots. B. Clarification of the derived subtypes, obtained through the aforementioned methodology, using scatter boxplots and heatmaps to depict the pairwise standardized effect size difference (Cohen’s d) between these subtypes.


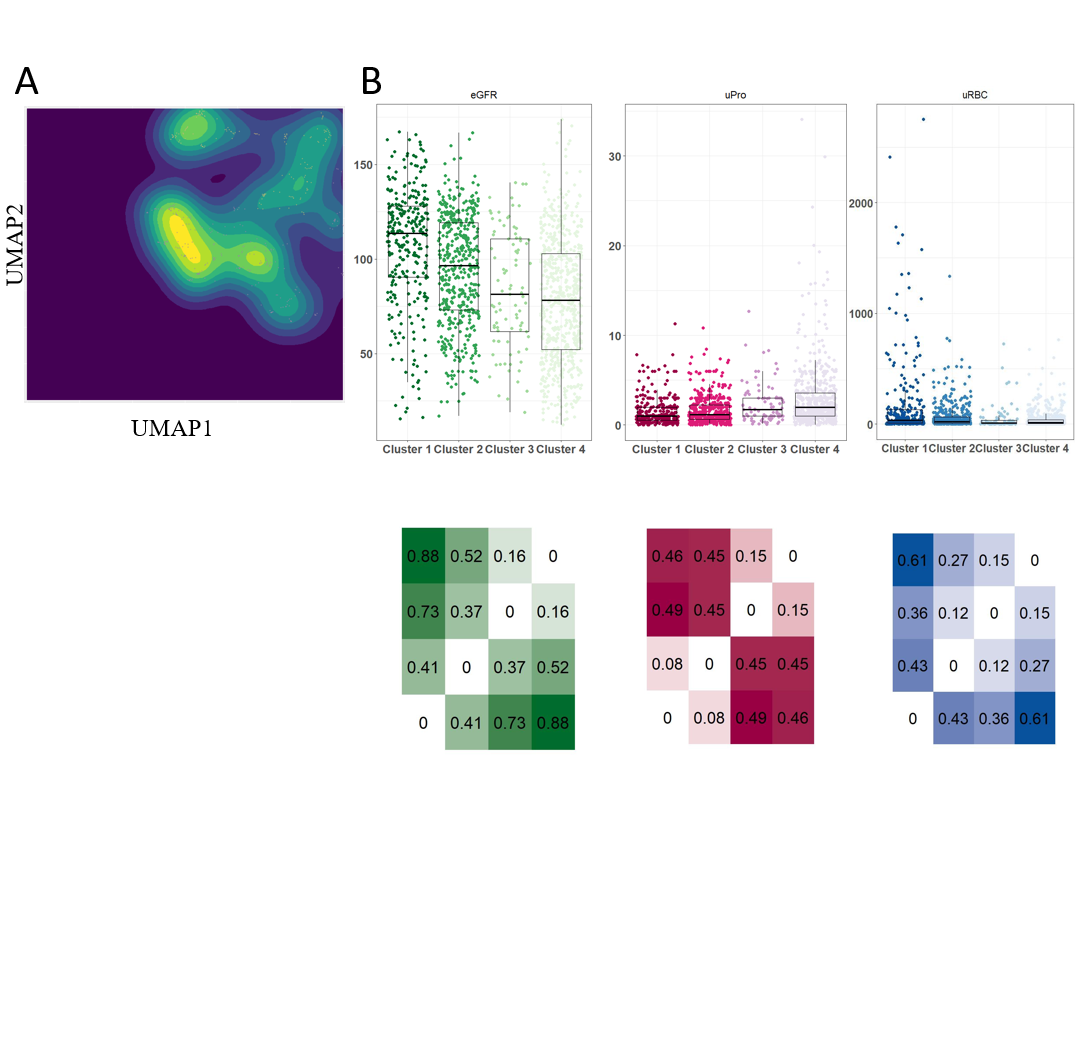


**Figure S4.** A. Visual representations of UMAP-reduced datasets integrated into **hierarchical clustering analyses utilizing network biomarkers**. Individuals are stratified by subtype and depicted through density plots. B. Clarification of the derived subtypes, obtained through the aforementioned methodology, using scatter boxplots and heatmaps to depict the pairwise standardized effect size difference (Cohen’s d) between these subtypes.


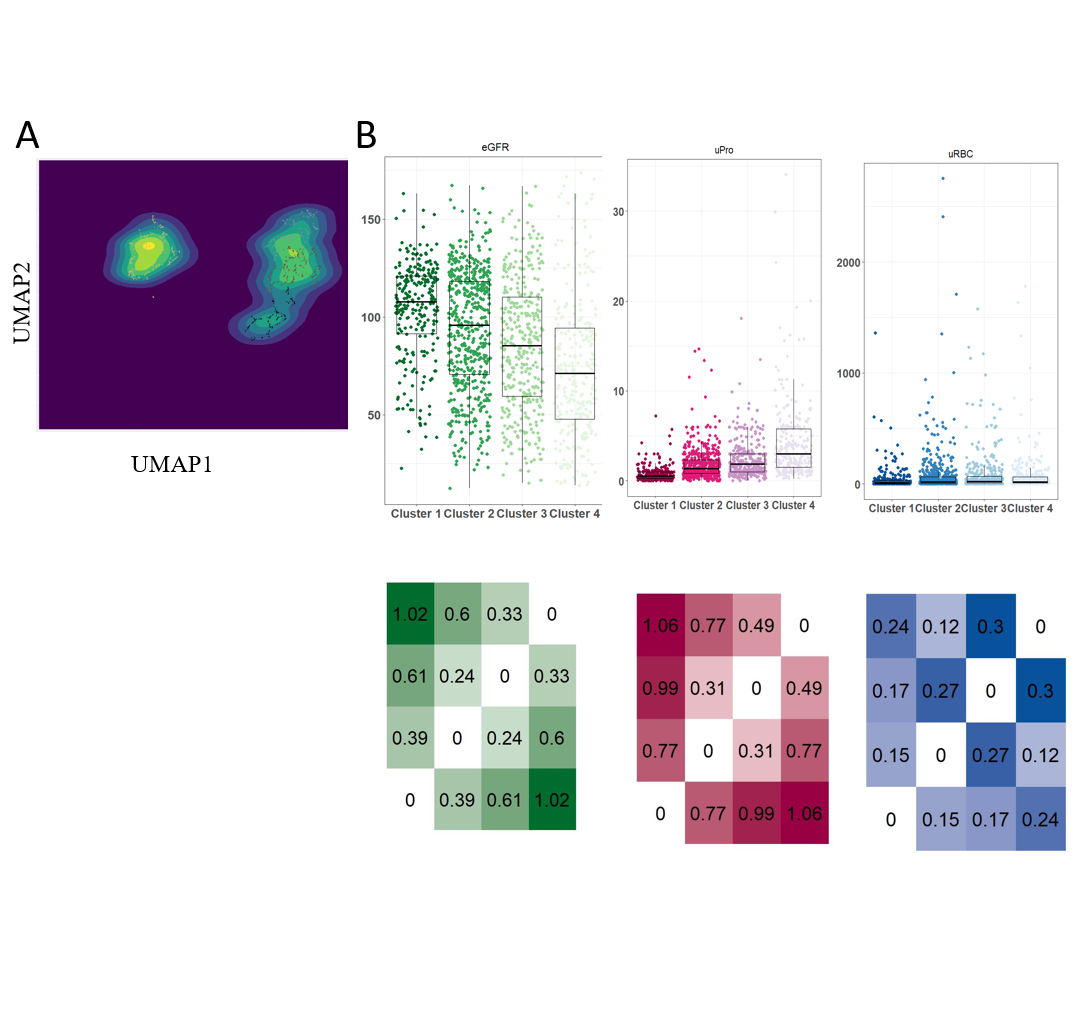


**Figure S5.** A. Kaplan–Meier curves depicting IgAN mortality, stratified by HCR grouping; B. Kaplan–Meier curves depicting IgAN mortality, stratified by KMA grouping; C. Forest plot visualization illustrating multi-factor Cox regression, stratified by HCR grouping; D. Forest plot visualization illustrating multi-factor Cox regression, stratified by KMA grouping; E. ROC curves representing the HCR grouping, with an AUC value of 0.71; F. ROC curves representing the KMA grouping, with an AUC value of 0.75.


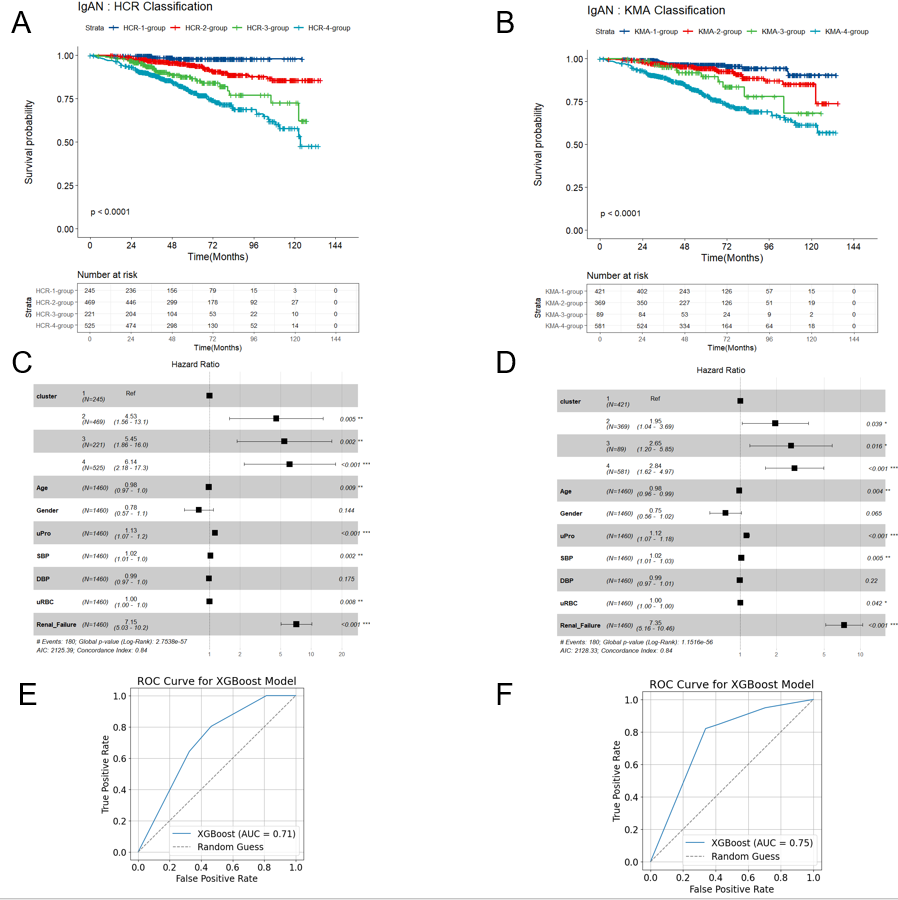


**Figure S6.** A. Sankey diagram illustrating KMN classification; B. Outcome distribution organized by KMN classification. All the results shows that cluster 1 has the best prognosis, whereas cluster 4 has the worst, consistent with clinical categorizations.


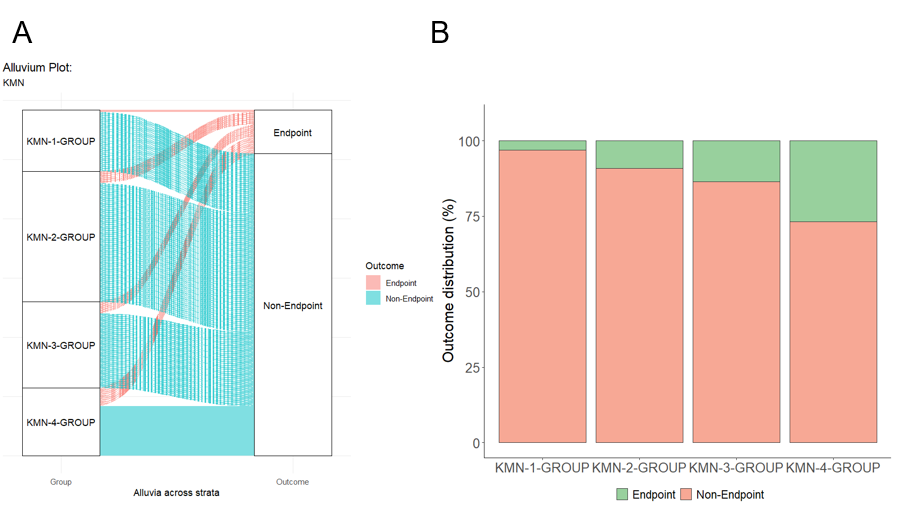


**Figure S7.** Violin plots illustrating the distribution of serum creatinine levels across distinct groups over various time intervals. The median value is denoted by the white point, the interquartile range is represented by the box range, the 95% confidence interval is depicted by the line range, and the width of the density plot reflects the frequency distribution. Panels include: A. Cluster 1 group; B. Cluster 2 group; C. Cluster 3 group; D. Cluster 4 group.


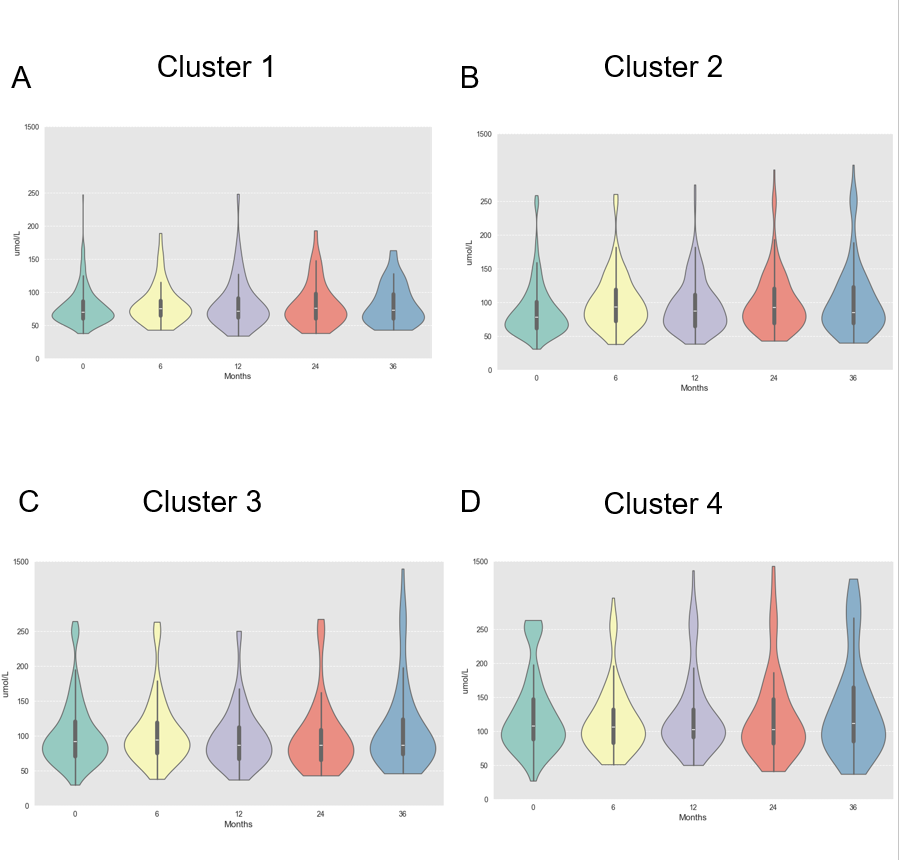


**Figure S8**. Violin plots illustrating the distribution of proteinuria levels across distinct groups over various time intervals. The median value is denoted by the white point, the interquartile range is represented by the box range, the 95% confidence interval is depicted by the line range, and the width of the density plot reflects the frequency distribution. Panels include: A. Cluster 1 group; B. Cluster 2 group; C. Cluster 3 group; D. Cluster 4 group.


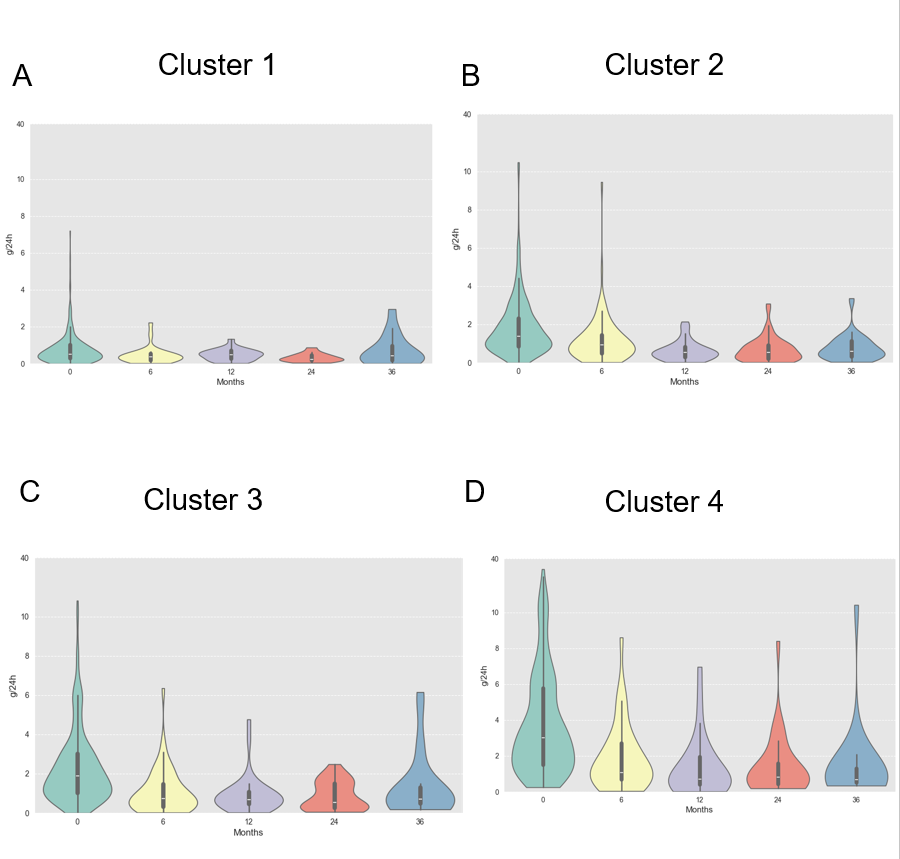


**Figure S9. A.** Forest plot visualization of multivariable Cox regression stratified by IIGAN grouping; **B.** Forest plot visualization of multivariable Cox regression stratified by RF-RG grouping.


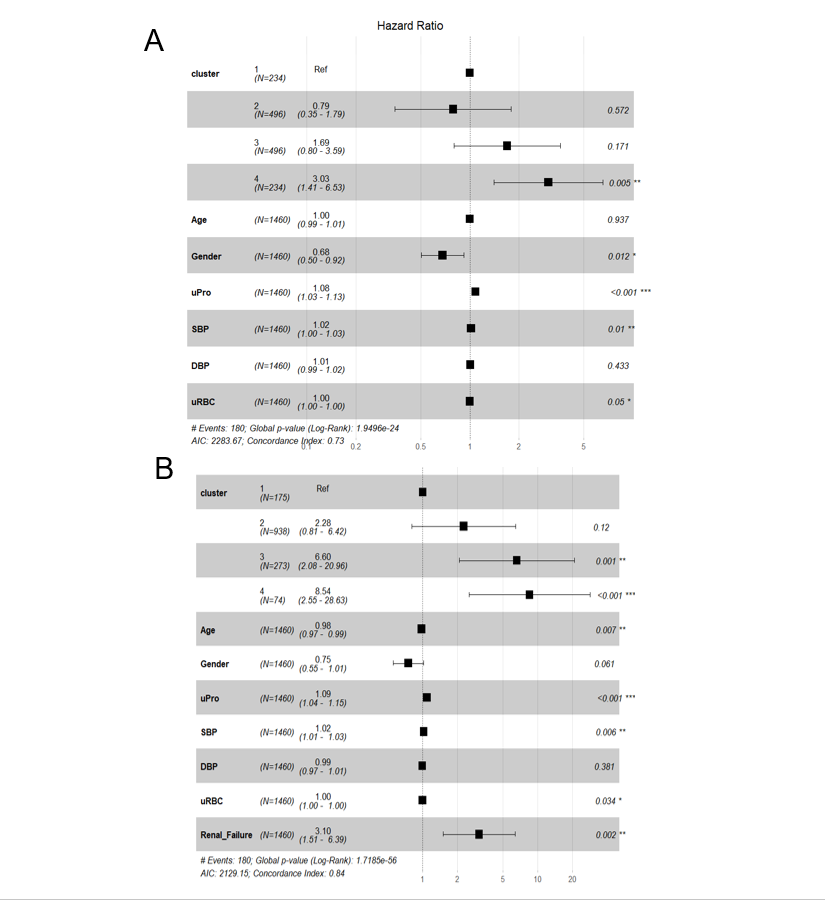


**Figure S10.** A. Outcome distribution organized by validation group; B. Sankey diagram illustrating validation group; C. Kaplan–Meier curves depicting IgAN mortality for the validation group.


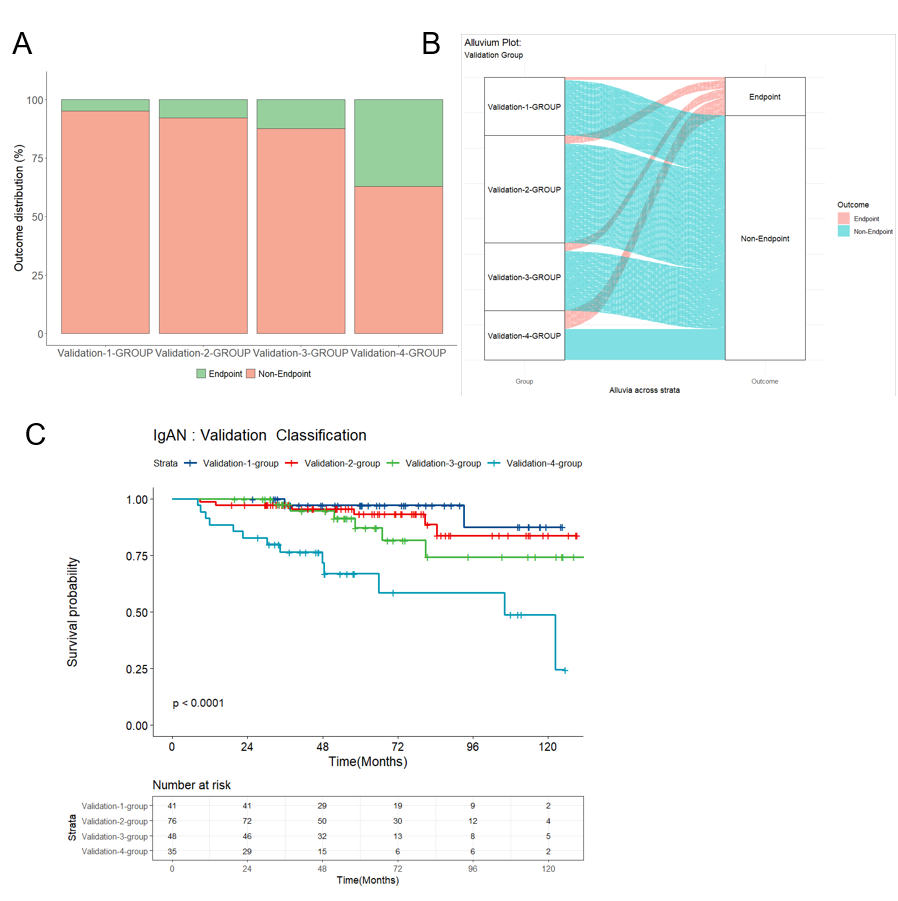


**Figure S11.** The forest plot visualization of multi-factor Cox regression, exploring the efficacy of immunosuppressive treatment or ACEI/ARB-based supportive care across different clusters. Panels include: A. Cluster 1, stratified by KMN clustering; B. Cluster 2; C. Cluster 4.


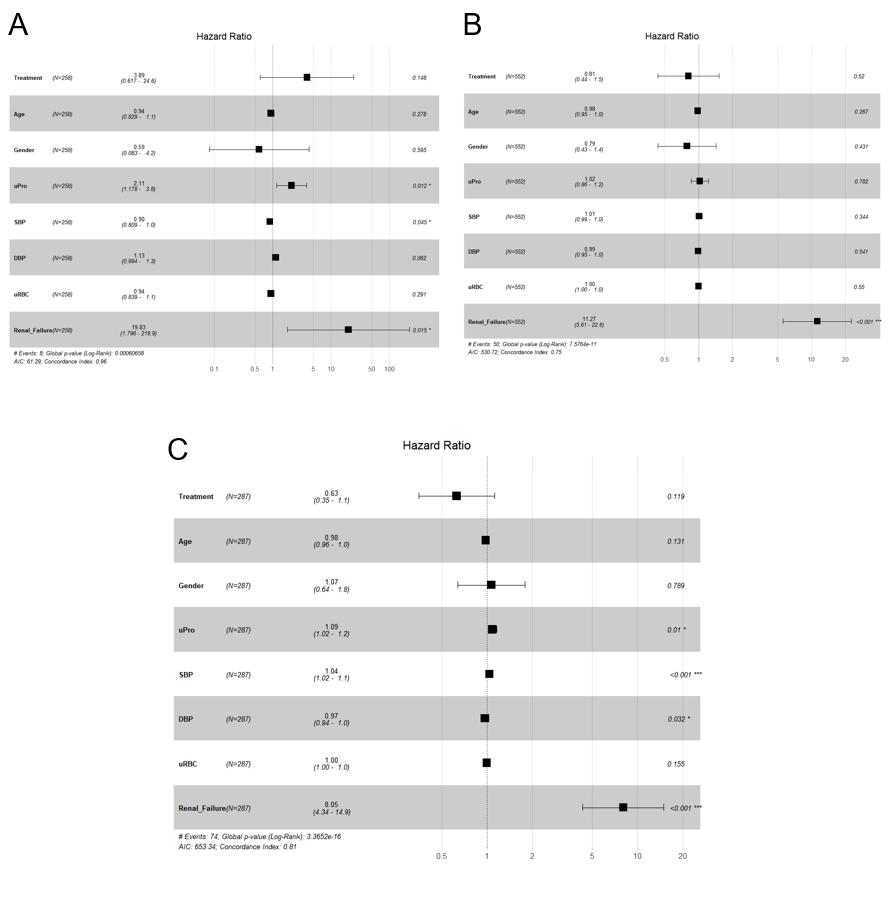


**Figure S12.** A. Prioritization of the top 10 indicators based on their mean absolute SHAP values, obtained from the k-means model using both renal and extra-renal indicators. Regions highlighted in red within the feature values indicate greater magnitudes. B. Prioritization of the top 10 indicators based on their mean absolute SHAP values, obtained from the k-means model using network biomarkers. Regions highlighted in red within the feature values indicate greater magnitudes.


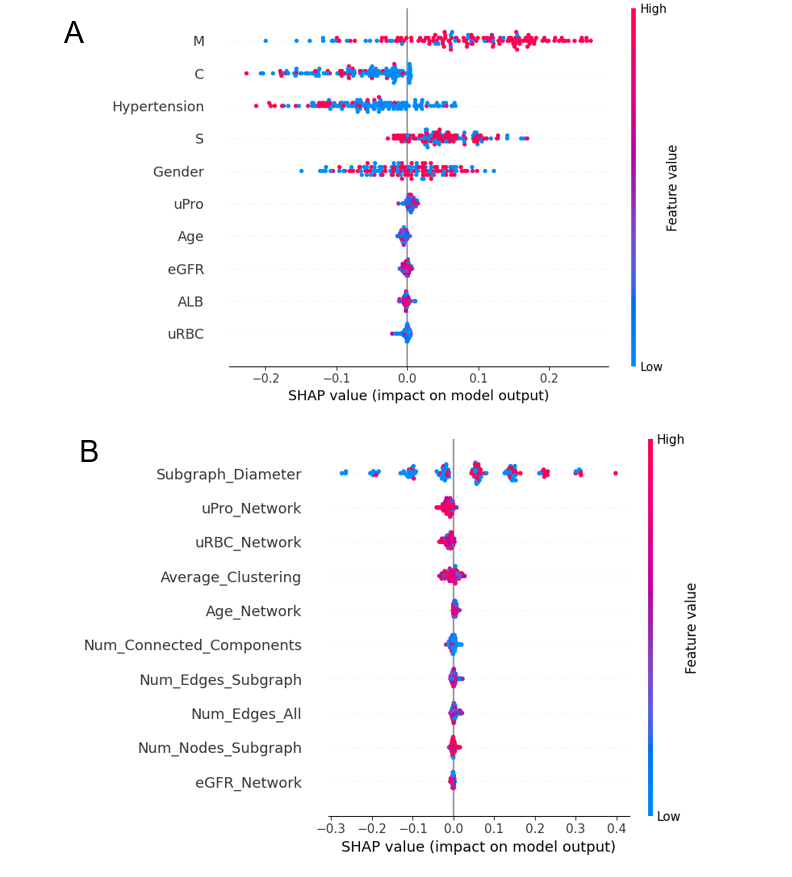


**Figure S13**. The primary network structure, rooted in Spearman correlation analysis for four distinct groups, with proteinuria as the focal point. This network includes indicators directly linked to proteinuria with statistical significance (p<0.05). Panels include: A. Cluster 1 group; B. Cluster 2 group; C. Cluster 3 group; D. Cluster 4 group.


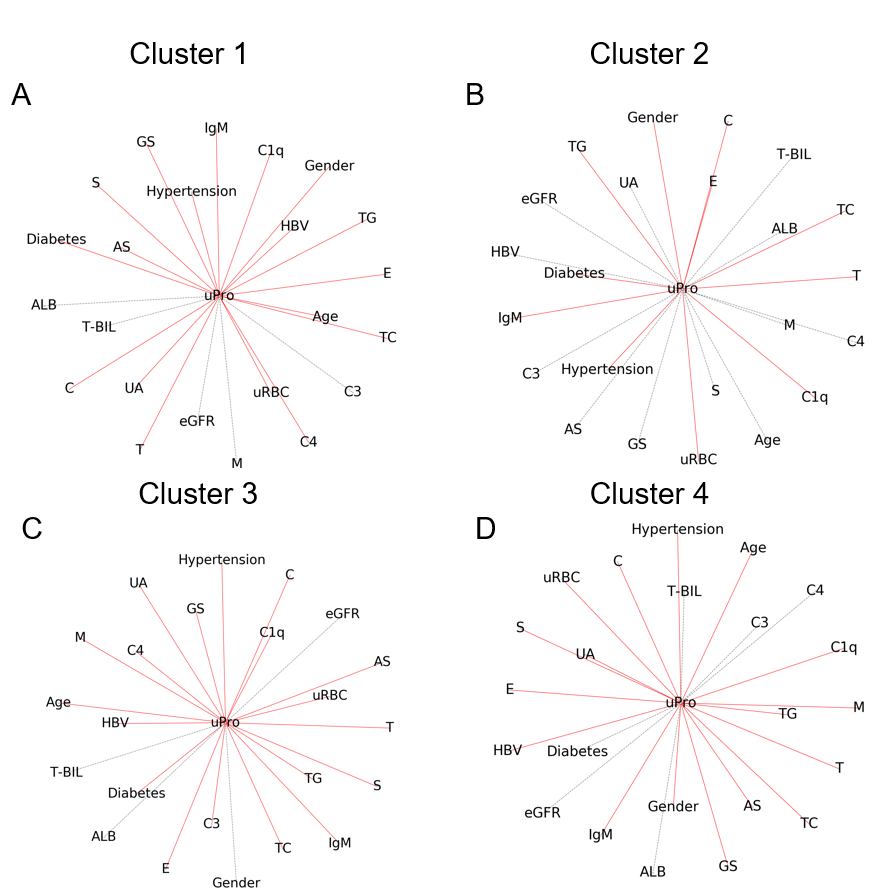


**Figure S14**. The primary network structure, rooted in Spearman correlation analysis for four distinct groups, with hematuria as the focal point. This network includes indicators directly linked to hematuria with statistical significance (p<0.05). Panels include: A. Cluster 1 group; B. Cluster 2 group; C. Cluster 3 group; D. Cluster 4 group.


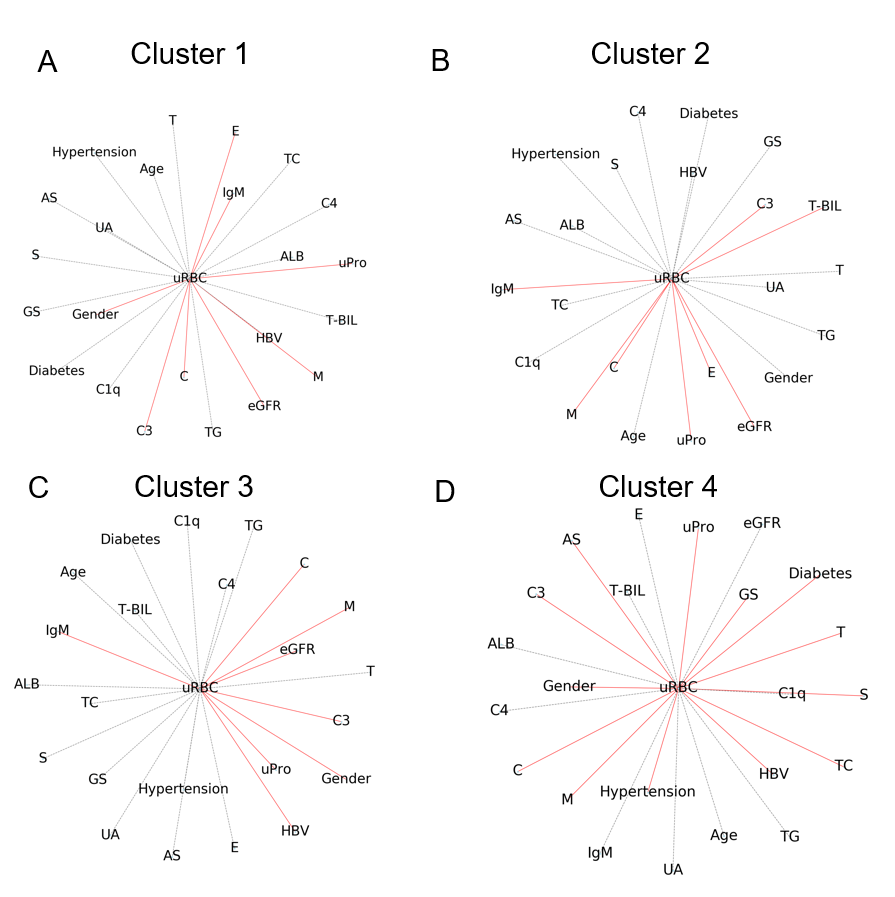


**Figure S15**. The primary network structure, rooted in Spearman correlation analysis for four distinct groups, with age as the focal point. This network includes indicators directly linked to age with statistical significance (p<0.05). Panels include: A. Cluster 1 group; B. Cluster 2 group; C. Cluster 3 group; D. Cluster 4 group.


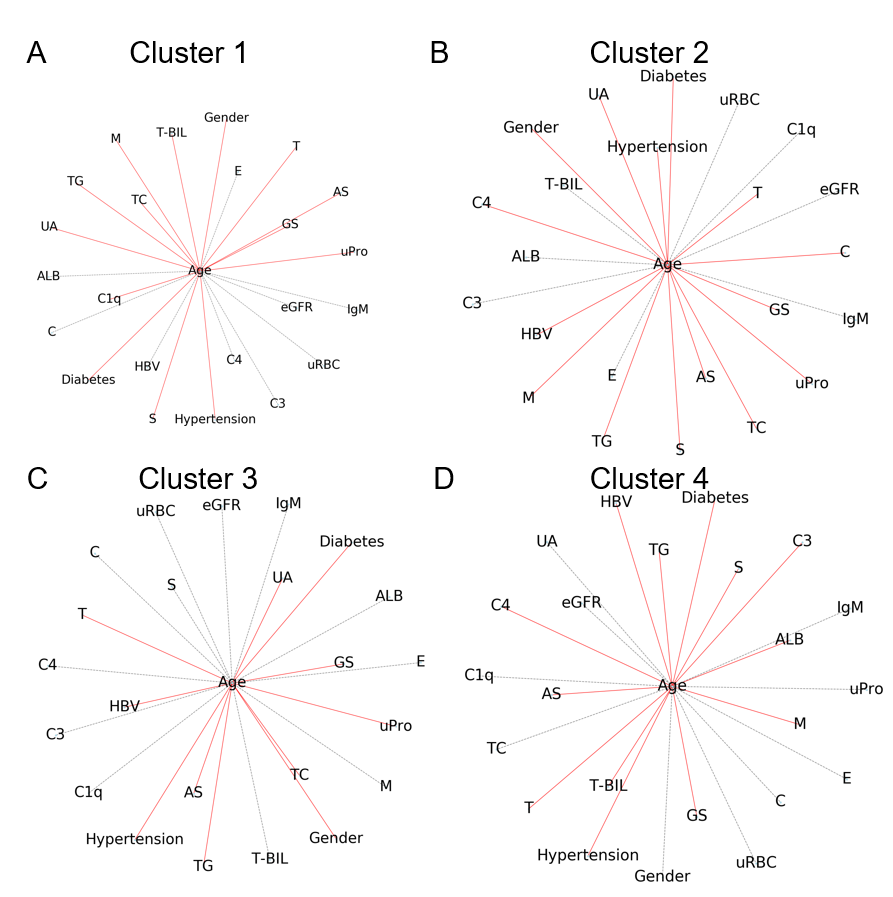


**Figure S16.** The primary network structure, rooted in Spearman correlation analysis for four distinct groups, with eGFR as the focal point. This network includes indicators directly linked to eGFR with statistical significance (p<0.05). Panels include: A. Cluster 1 group; B. Cluster 2 group; C. Cluster 3 group; D. Cluster 4 group.


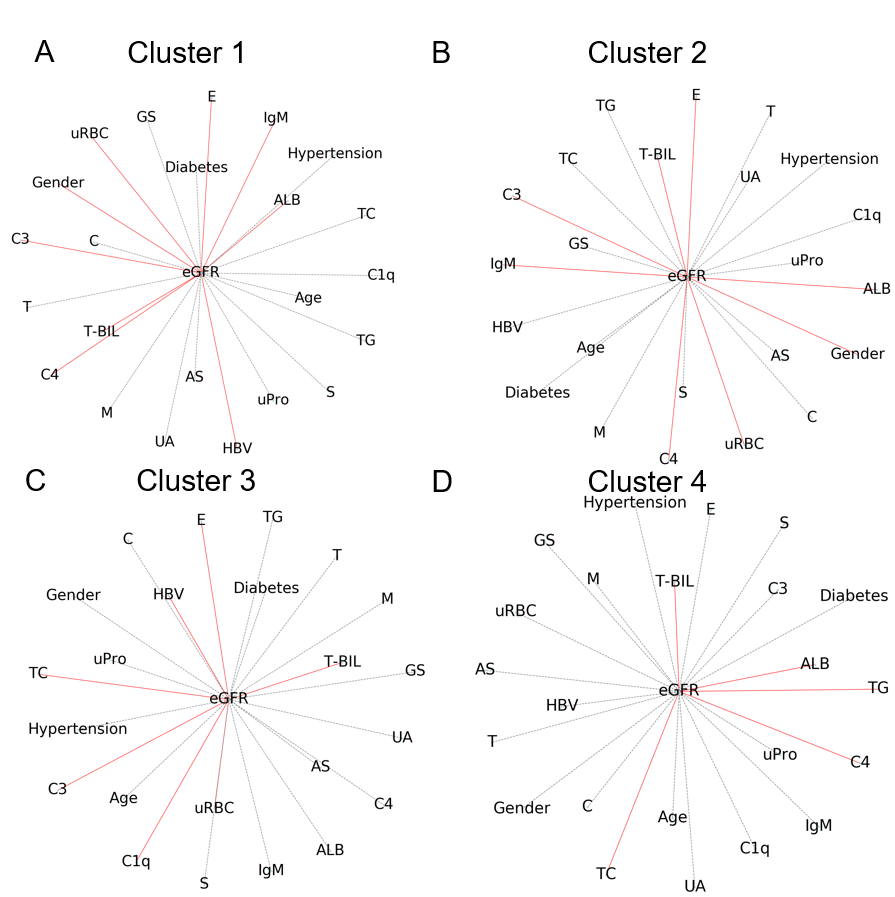


**Figure S17**. Correlation networks of serum metabolomics data in Clusters 1 to 4. Nodes represent individual metabolite data, while edges indicate statistically significant differences (p<0.05) via Spearman correlation among metabolites. Edge styles, represented by dashed and solid lines, denote negative and positive correlations, respectively.


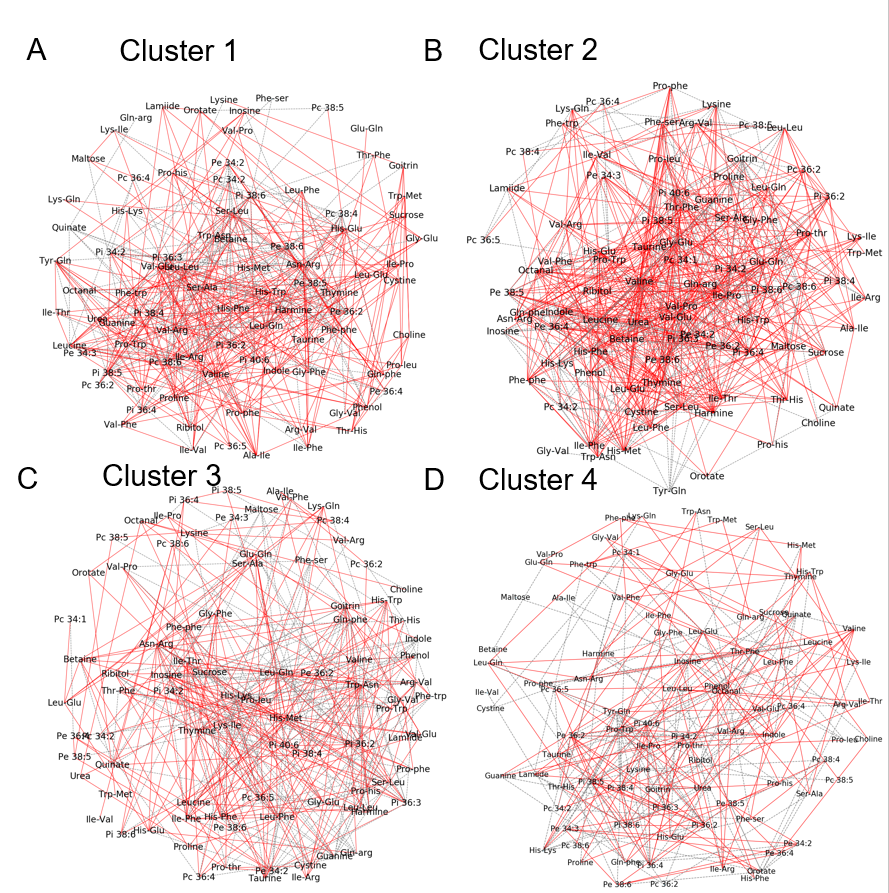


**Figure S18.** A. Heatmap illustrating the correlations among serum metabolites as well as their associations with clinical manifestations. B. Heatmap showing the interrelations among gut microbiota components and their correlations with clinical symptoms. Red indicates a positive correlation, while blue denotes a negative correlation. Curve plots demonstrate the statistical associations between the corresponding metabolites or microbiota and clinicopathological features. The width of each curve indicates the strength of the correlation, with P values highlighted to denote statistical significance.


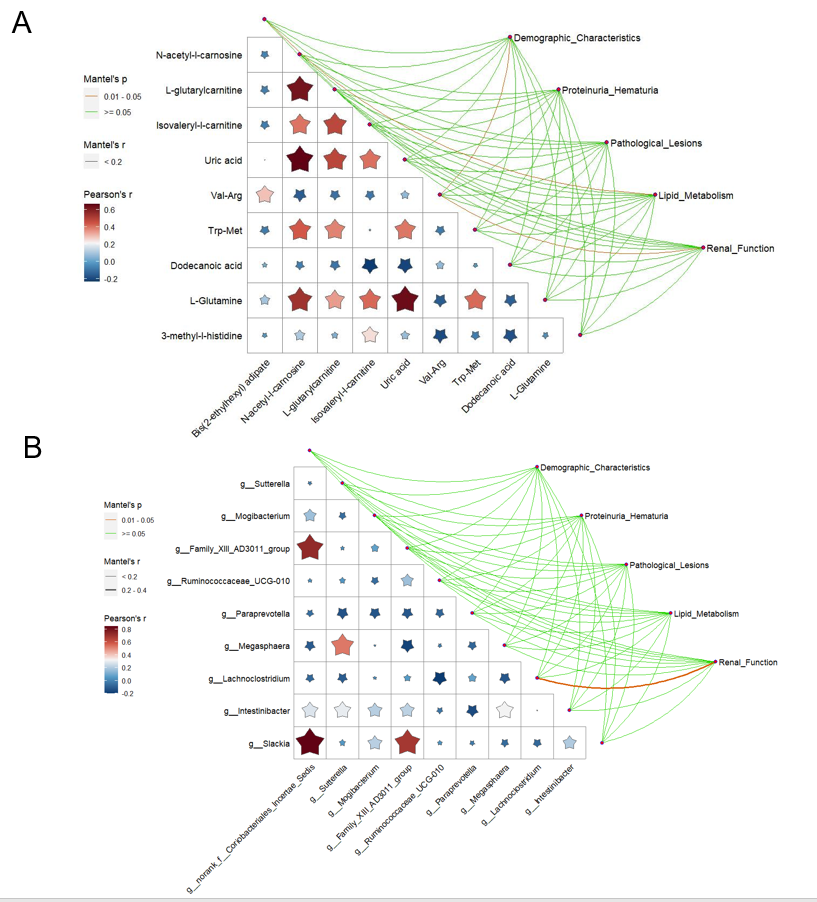


**Figure S19.** The network architecture, derived from the correlation analysis, centers around Bis(2-ethylhexyl) adipate as the focal node, encompassing metabolites directly interconnected with it.


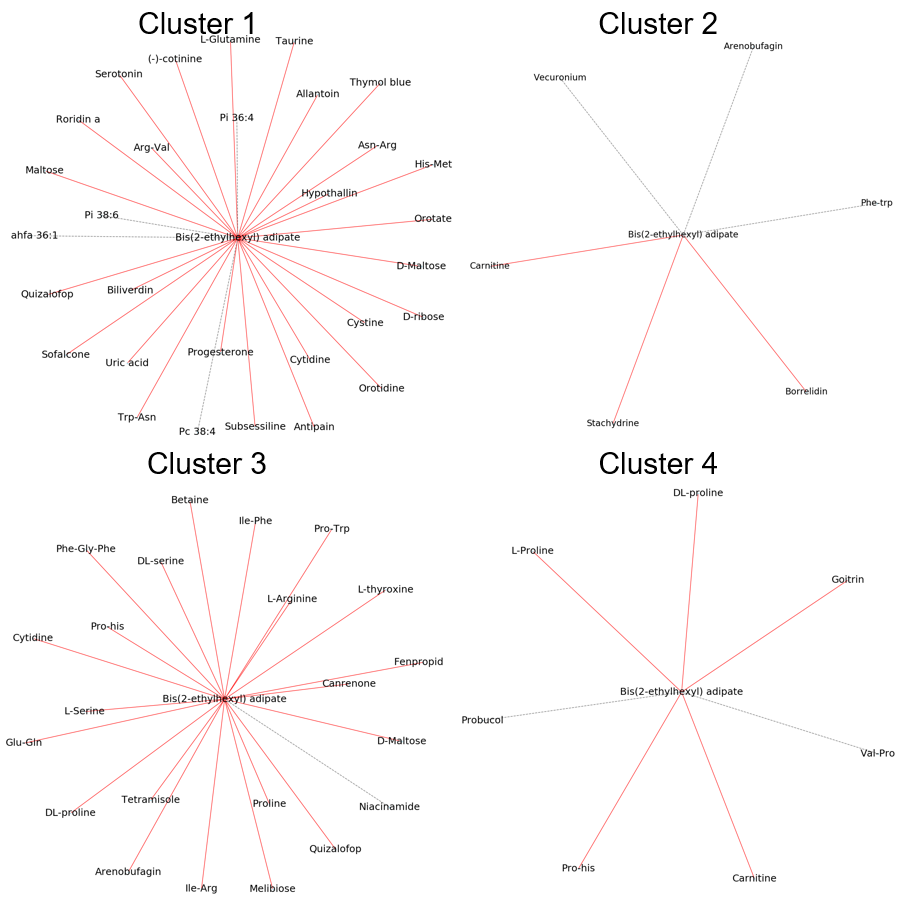


**Figure S20.** The network architecture, derived from the correlation analysis, centers around Dodecanoic acid as the focal node, encompassing metabolites directly interconnected with it.


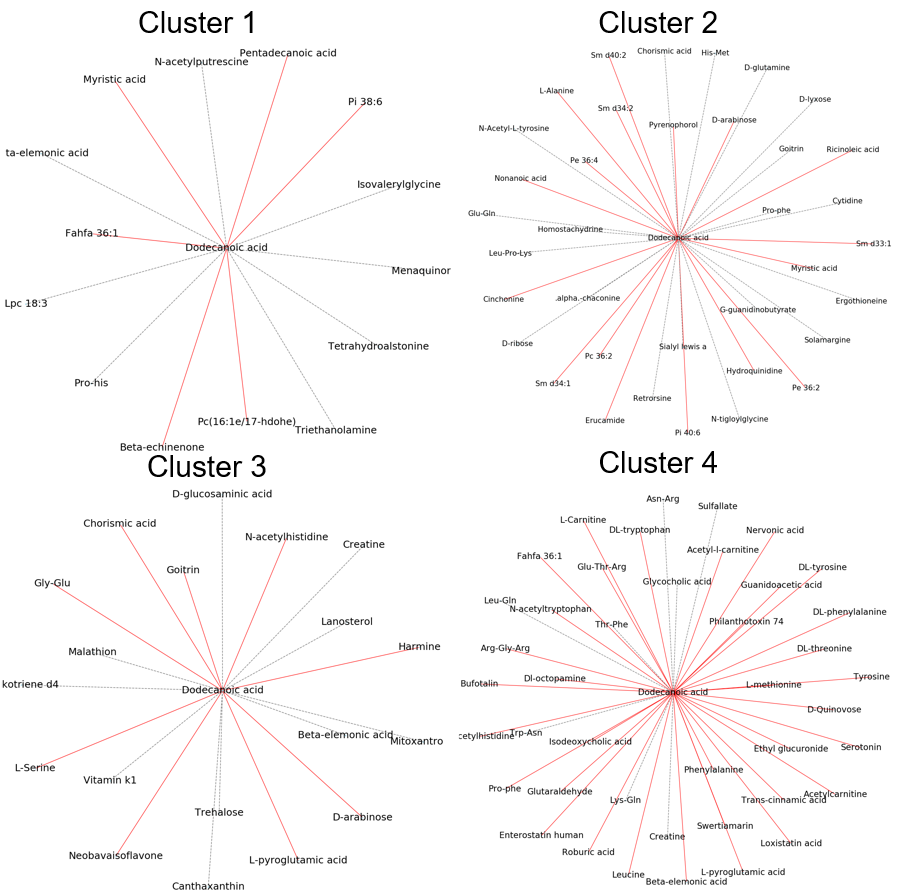


**Figure S21.** The network architecture, derived from the correlation analysis, centers around N-acetyl-l-carnosine as the focal node, encompassing metabolites directly interconnected with it.


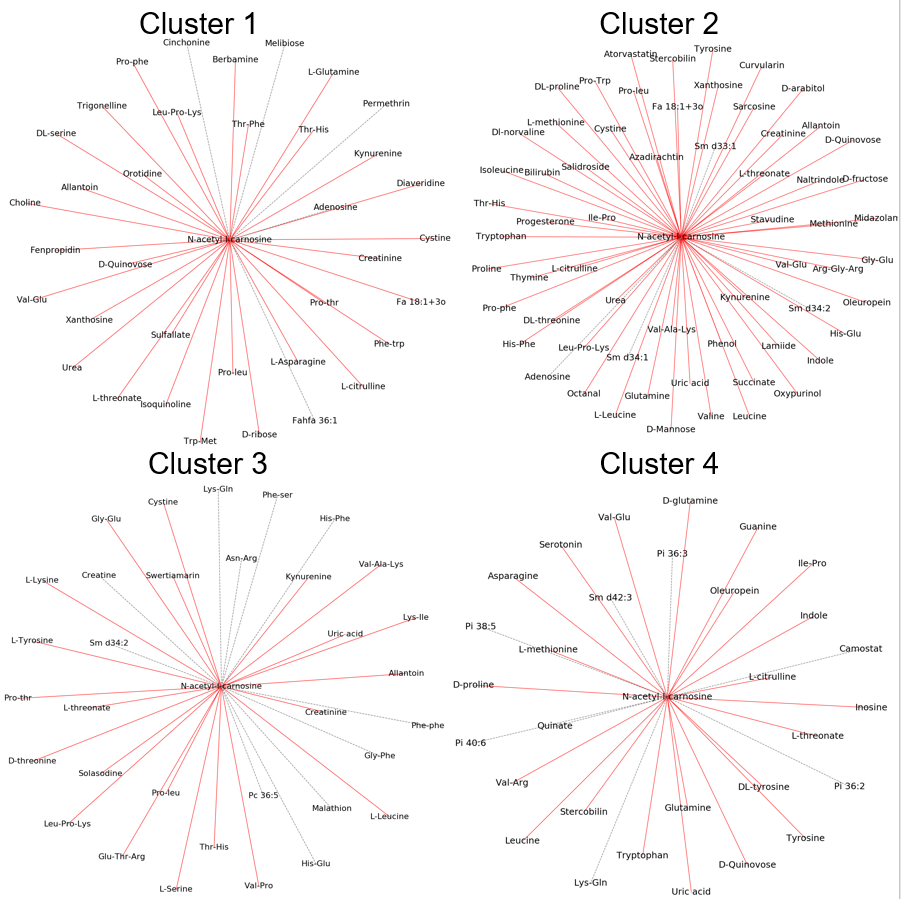


**Figure S22.** The network architecture, derived from the correlation analysis, centers around L-glutarylcarnitine as the focal node, encompassing metabolites directly interconnected with it.


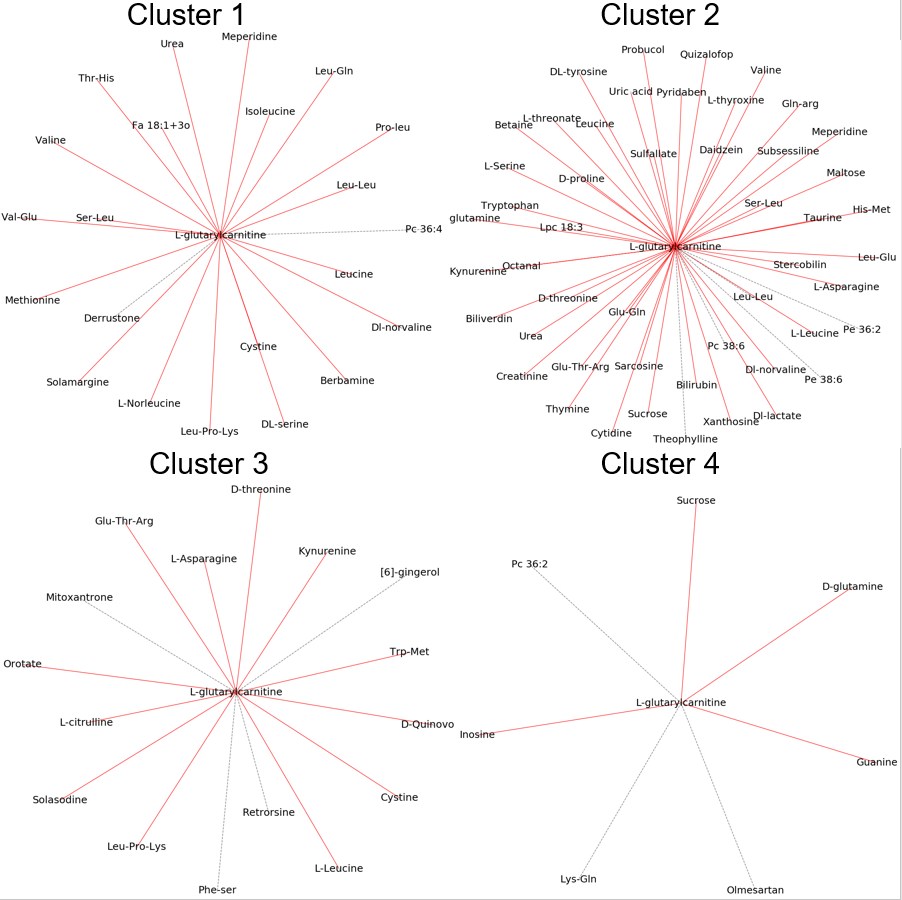


**Figure S23.** The network architecture, derived from the correlation analysis, centers around Isovaleryl-l-carnitine as the focal node, encompassing metabolites directly interconnected with it.


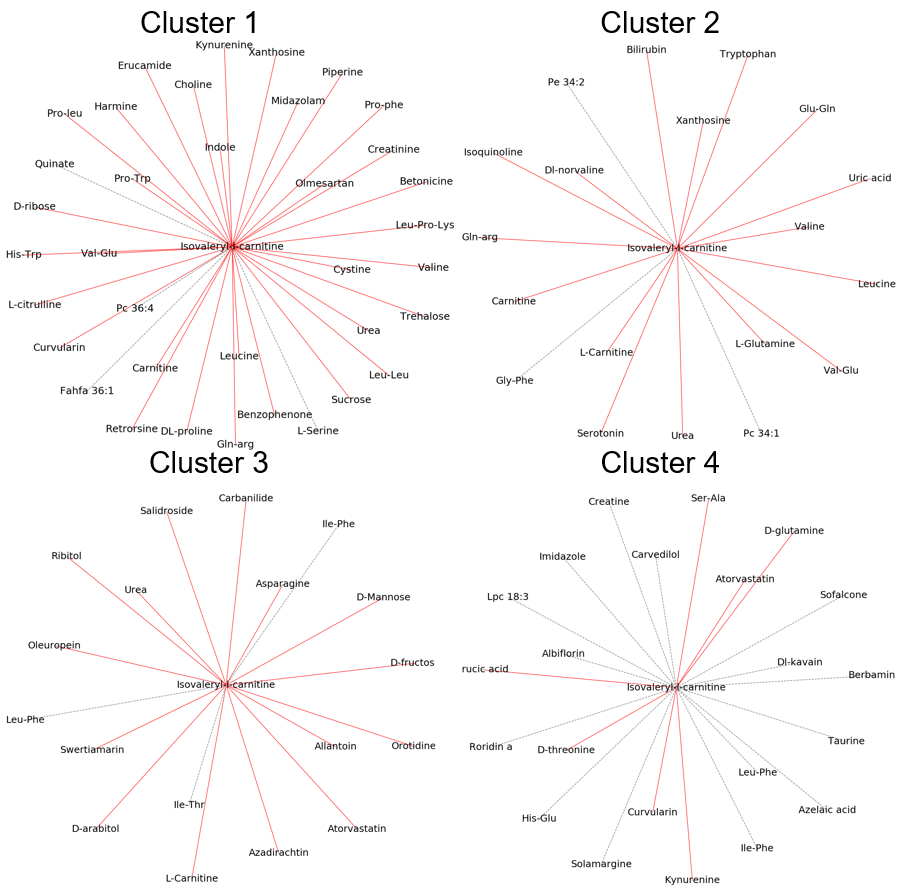


**Figure S24.** The network architecture, derived from the correlation analysis, centers around uric acid, as the focal node, encompassing metabolites directly interconnected with it.


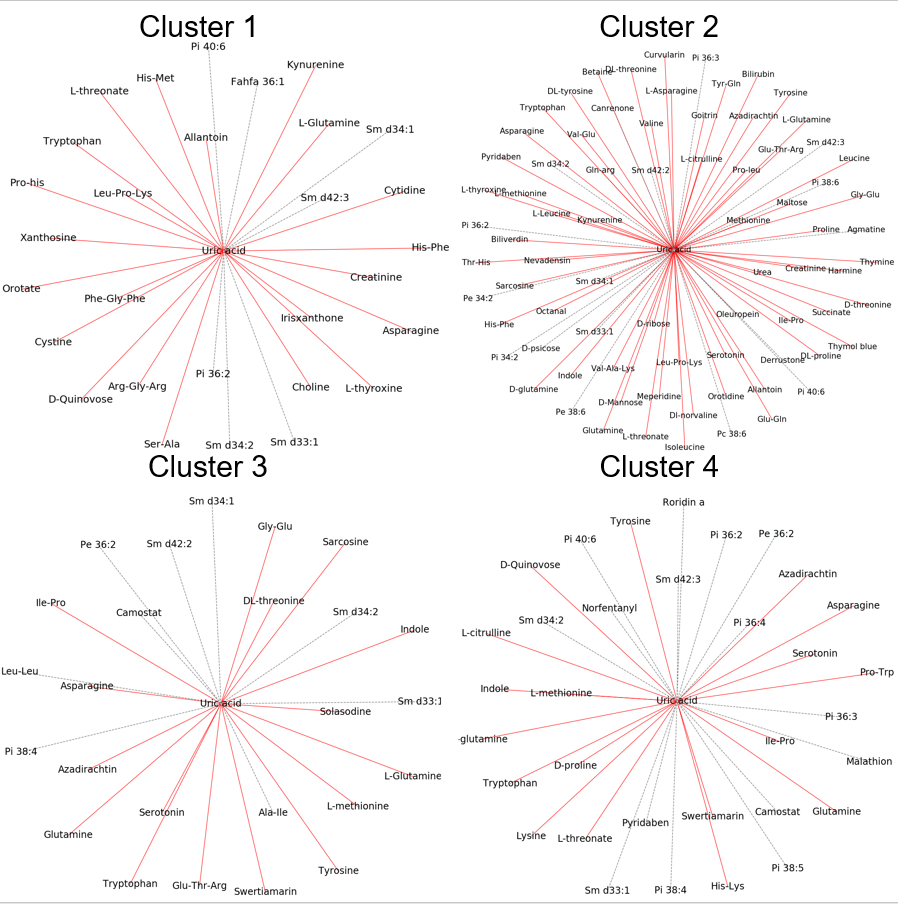


**Figure S25.** The network architecture, derived from the correlation analysis, centers around 3-methyl-l-histidine, as the focal node, encompassing metabolites directly interconnected with it.


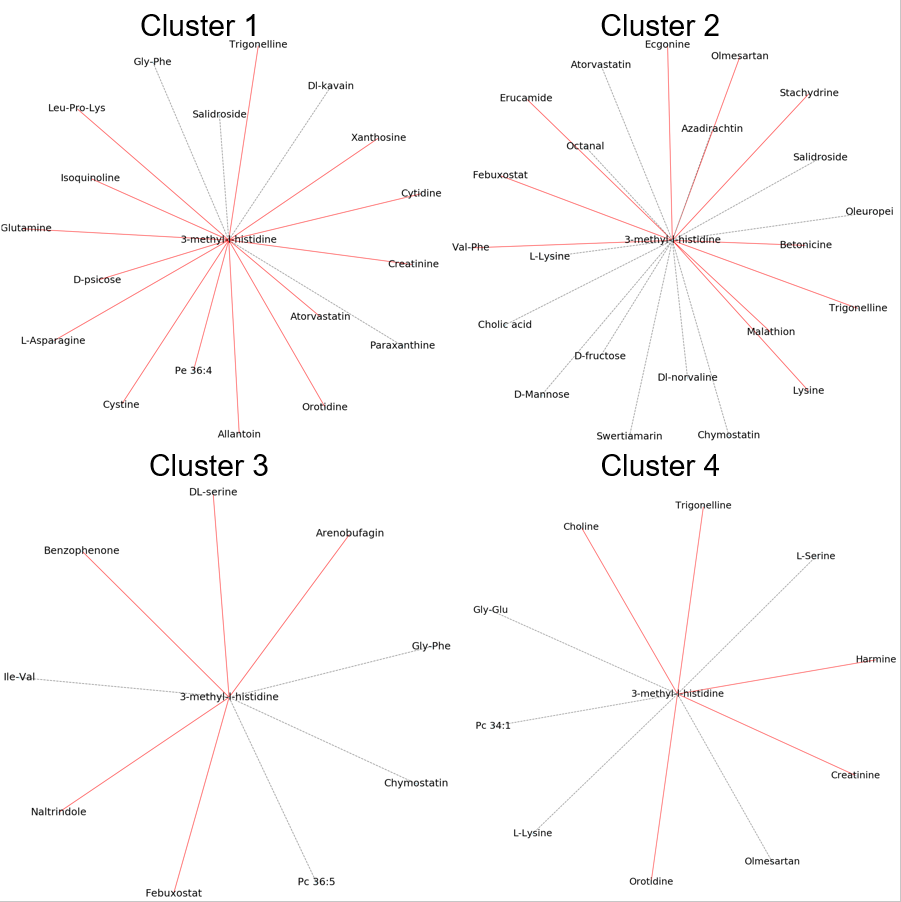


**Figure S26.** illustrates correlation networks of gut microbiota data in Clusters 1 to 4. Nodes represent individual metabolite data, while edges indicate statistically significant differences (p<0.05) via Spearman correlation among gut microbiota. Edge styles, represented by dashed and solid lines, denote negative and positive correlations, respectively.


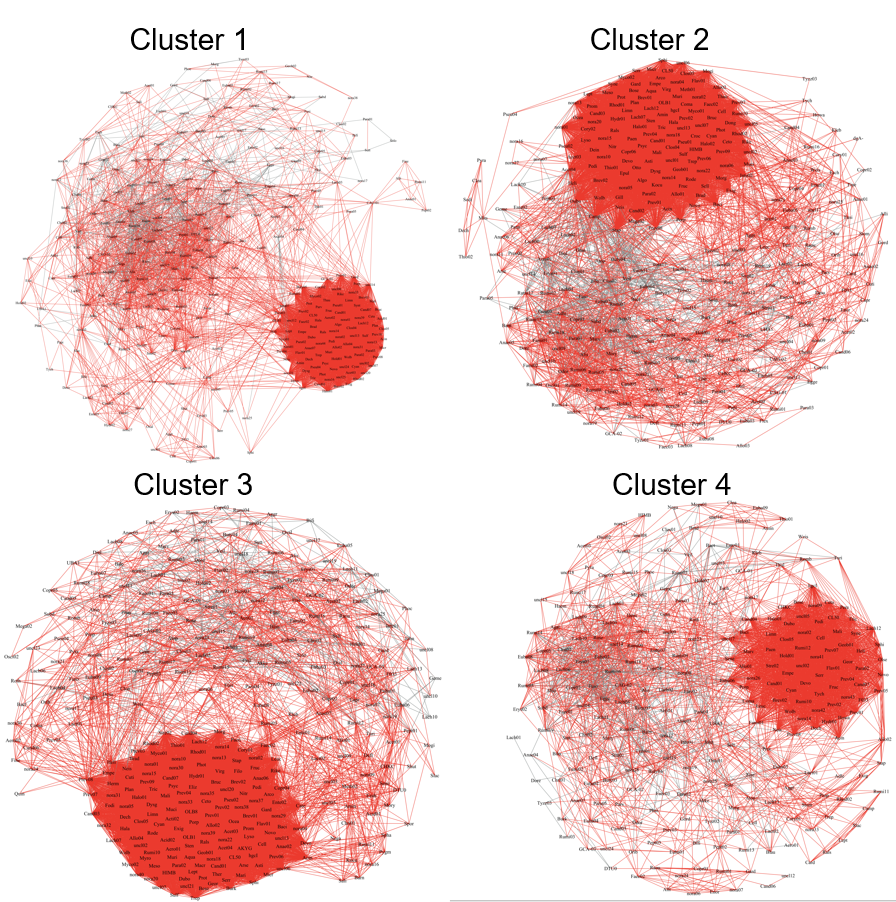


**Figure S27.** The network architecture, derived from the correlation analysis, centers around Paraprevotella, as the focal node, encompassing metabolites directly interconnected with it.


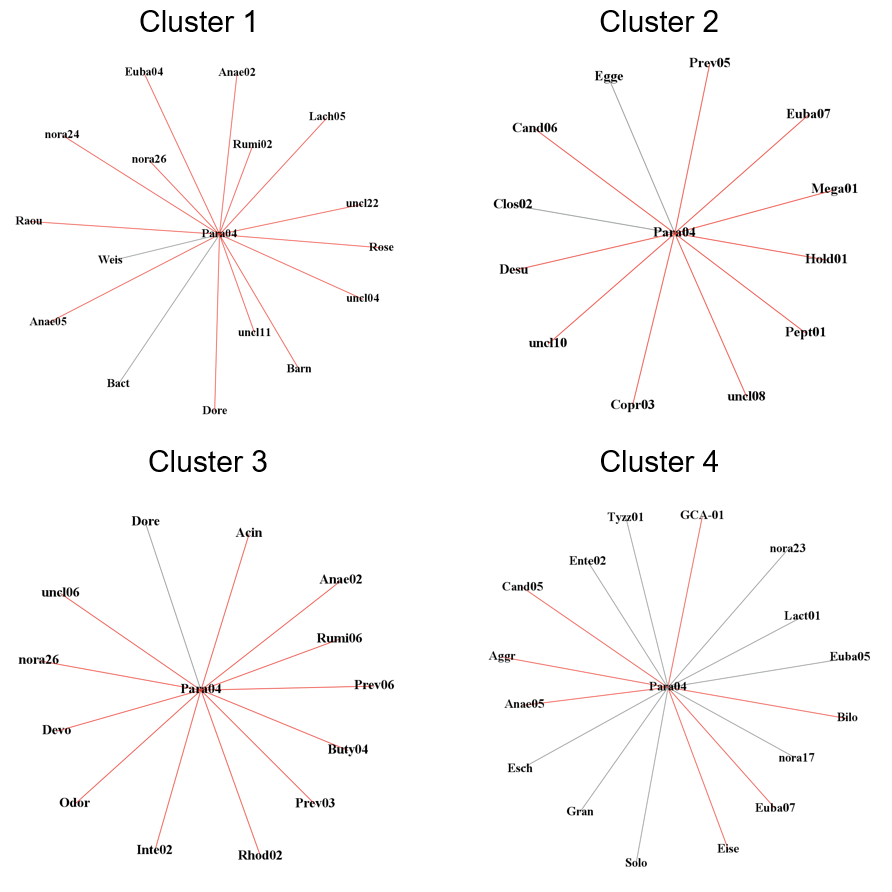


**Figure S28.** The network architecture, derived from the correlation analysis, centers around Lachnoclostridium, as the focal node, encompassing metabolites directly interconnected with it.


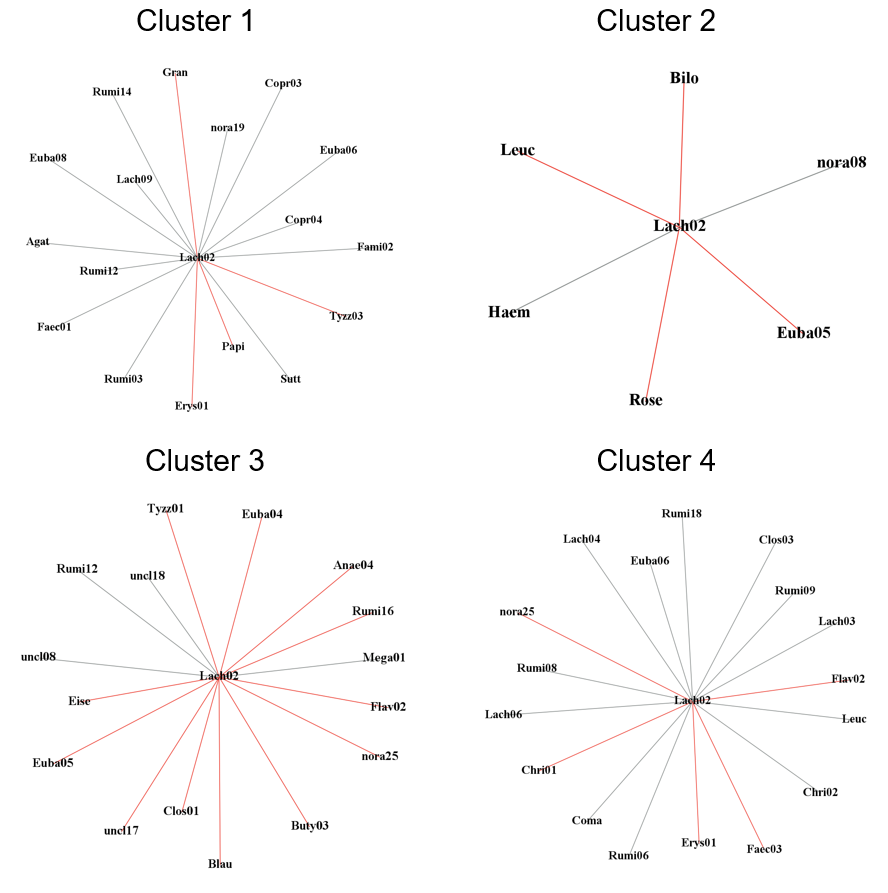


**Figure S29.** The network architecture, derived from the correlation analysis, centers around norank_f_Coriobacteriales_Incertae_Sedis, as the focal node, encompassing metabolites directly interconnected with it.


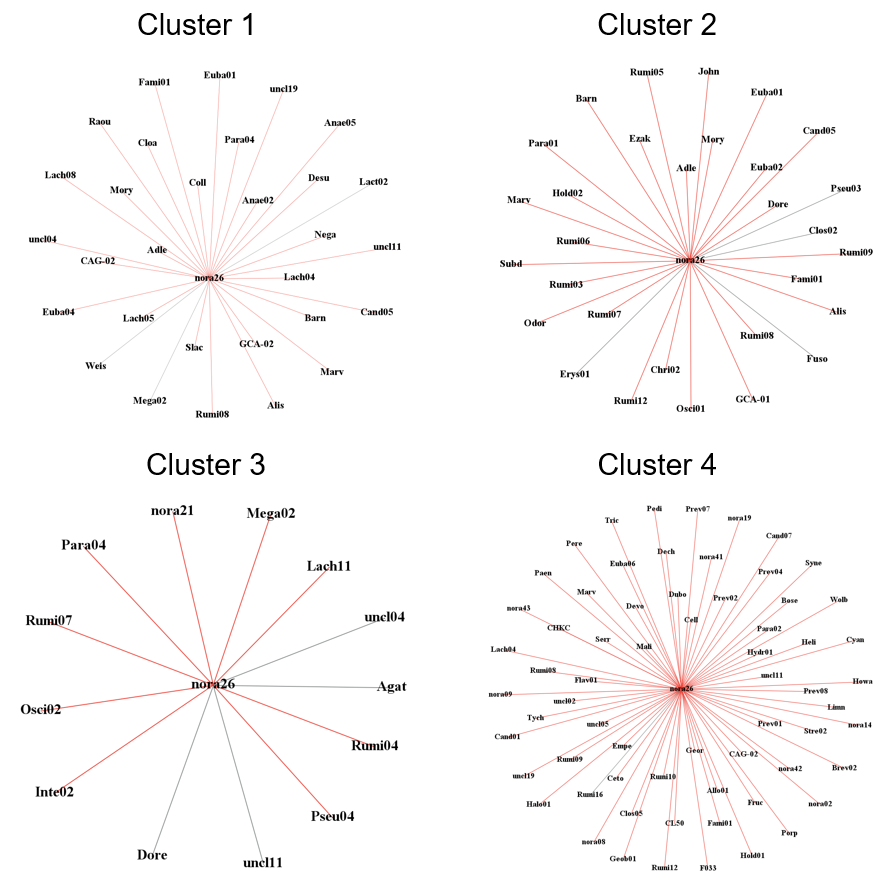


**Figure S30.** The network architecture, derived from the correlation analysis, centers around Sutterella, as the focal node, encompassing metabolites directly interconnected with it.


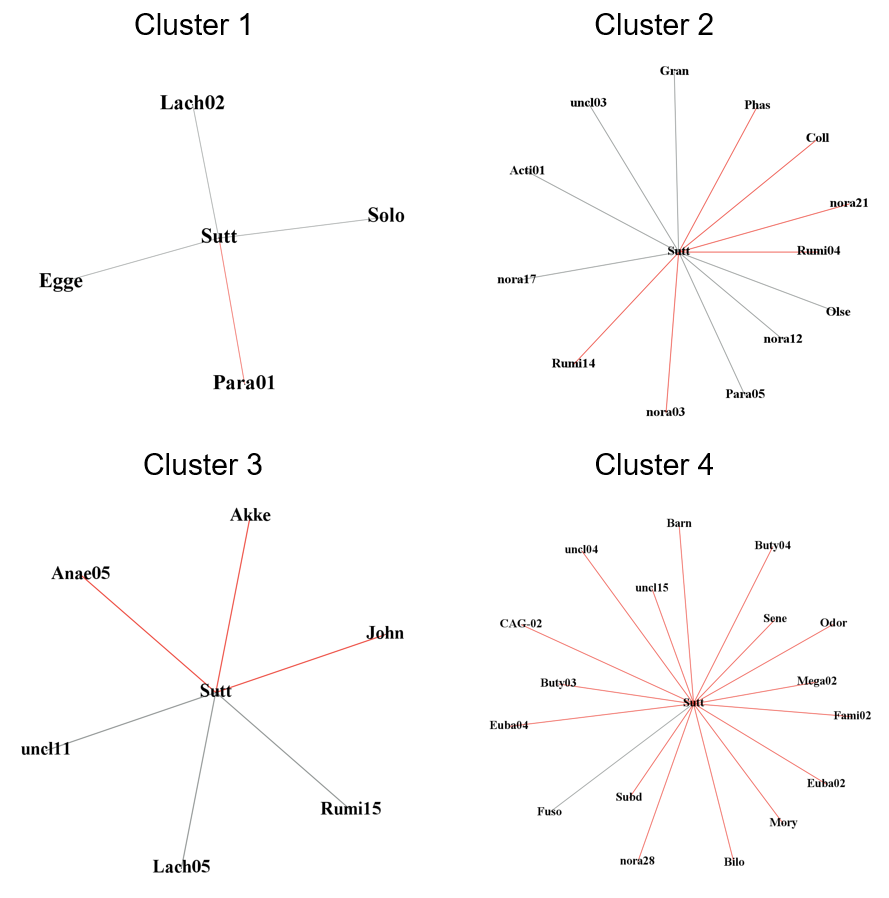


**Figure S31.** The network architecture, derived from the correlation analysis, centers around Family_XI AD3011_group, as the focal node, encompassing metabolites directly interconnected with it.


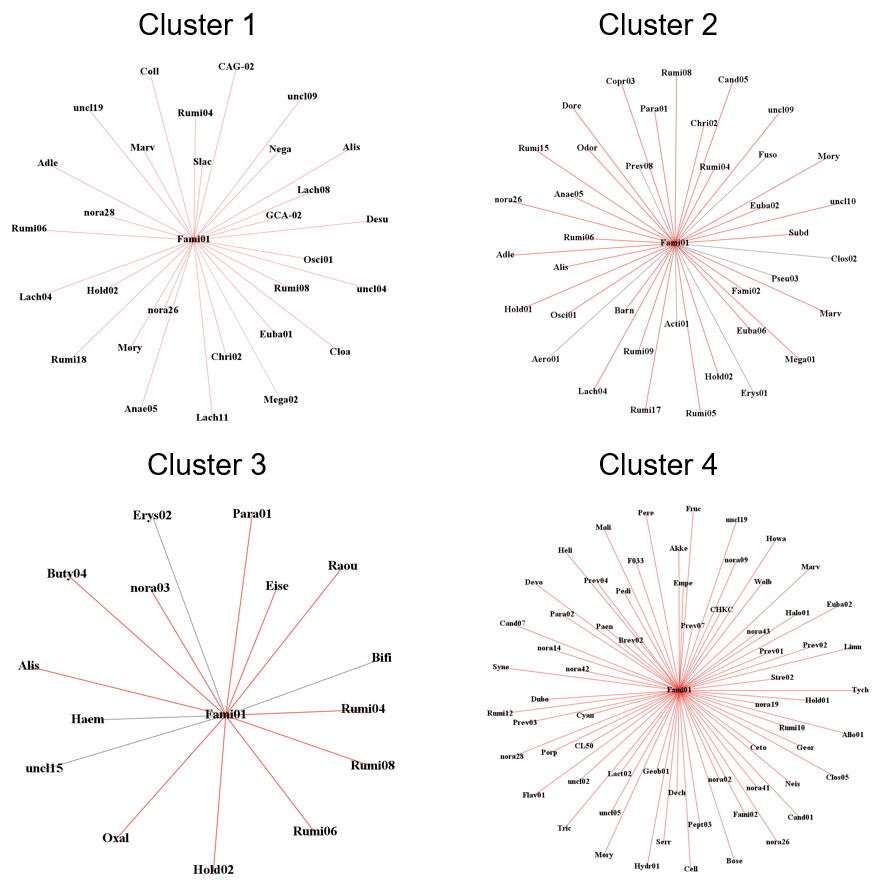


**Figure S32.** The network architecture, derived from the correlation analysis, centers around Ruminococcaceae_UCG-010, as the focal node, encompassing metabolites directly interconnected with it.


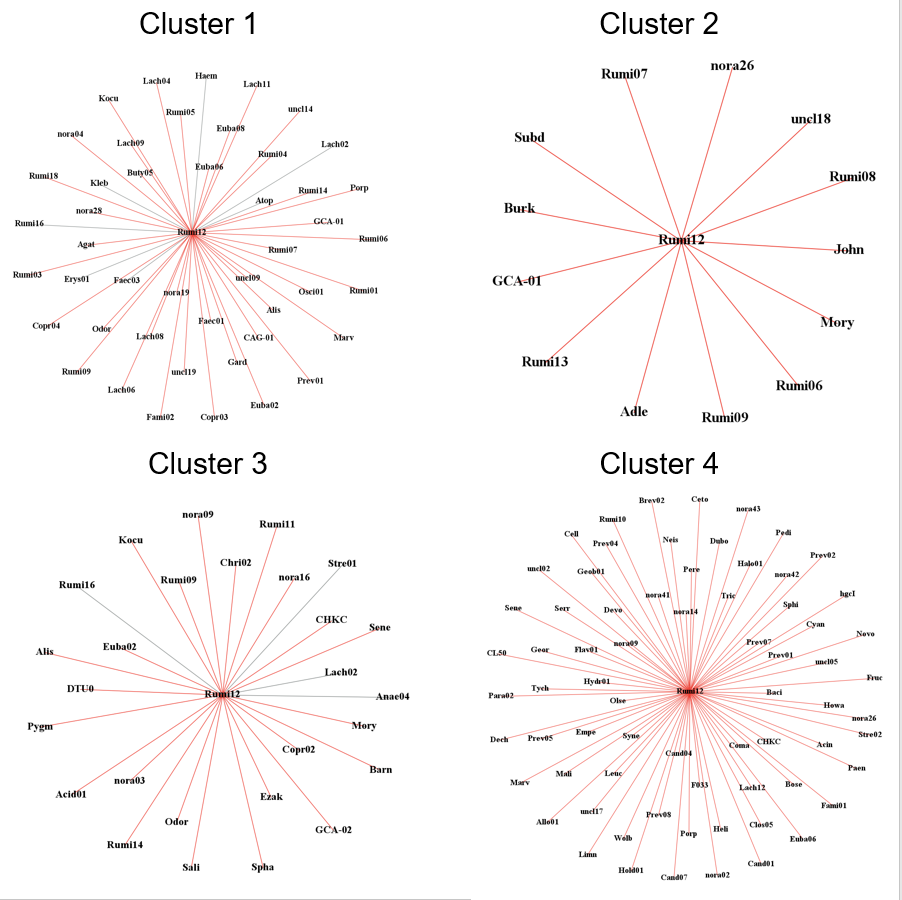


**Figure S33.** The network architecture, derived from the correlation analysis, centers around Megasphaera, as the focal node, encompassing metabolites directly interconnected with it.


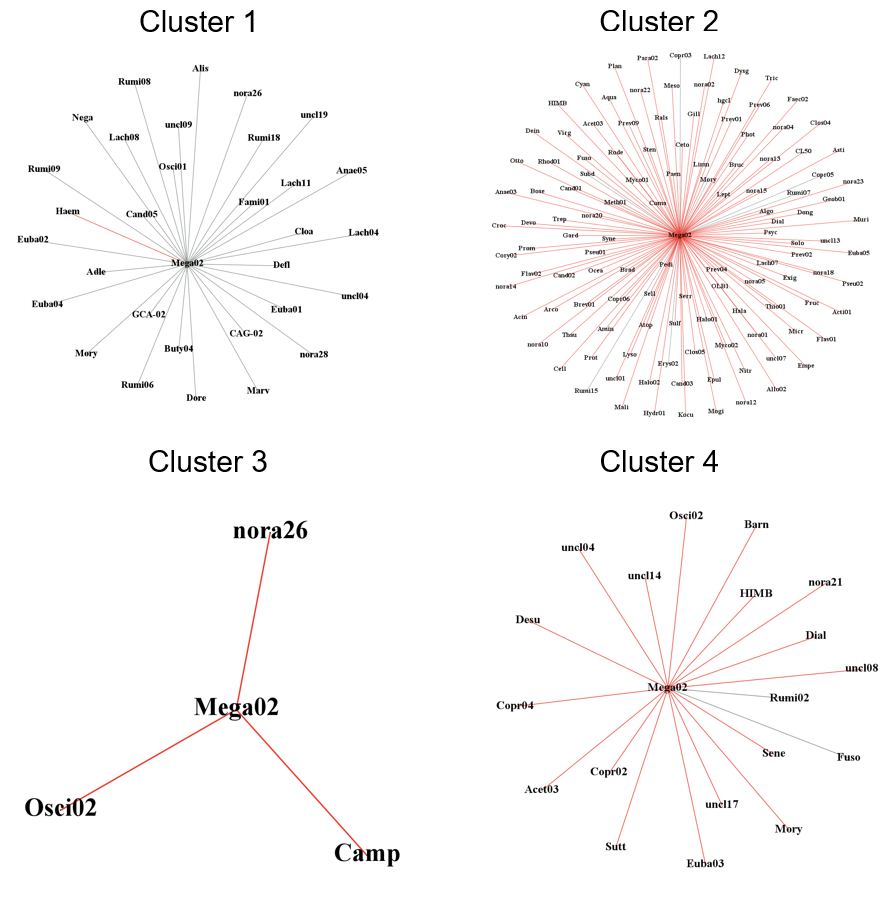


**Figure S34.** The network architecture, derived from the correlation analysis, centers around Intestinibaeter, as the focal node, encompassing metabolites directly interconnected with it.


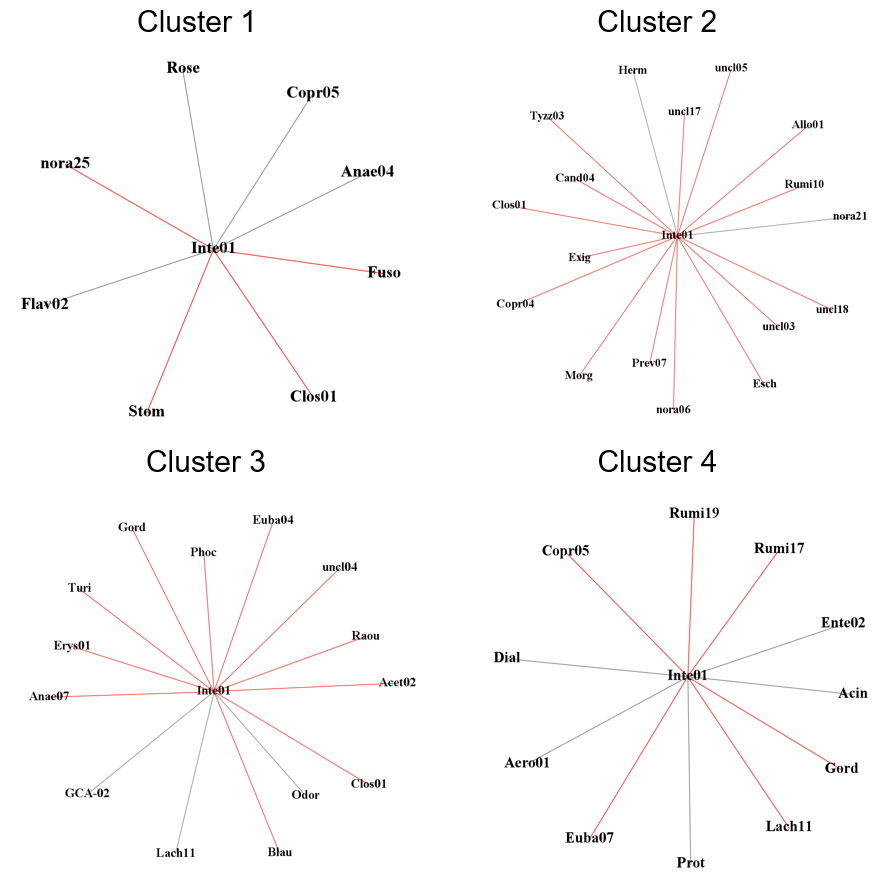

Supplement: Multimedia Appendix 1 [file jmir_v27i1e65563_app1.docx]
